# Glucose counteracts wood-dependent induction of lignocellulolytic enzyme secretion in monokaryon and dikaryon submerged cultures of the white-rot basidiomycete *Pleurotus ostreatus*

**DOI:** 10.1038/s41598-020-68969-1

**Published:** 2020-07-24

**Authors:** Manuel Alfaro, Andrzej Majcherczyk, Ursula Kües, Lucía Ramírez, Antonio G. Pisabarro

**Affiliations:** 1 Genetics, Genomics and Microbiology Research Group, Institute for Multidisciplinary Research in Applied Biology (IMAB-UPNa), Public University of Navarre, 31006 Pamplona, Spain; 2 Molecular Wood Biotechnology and Technical Mycology, Büsgen-Institute University of Goettingen, Büsgenweg 2, 37077 Göttingen, Germany

**Keywords:** Proteomics, Fungal biology

## Abstract

The secretome complexity and lignocellulose degrading capacity of *Pleurotus ostreatus* monokaryons mkPC9 and mkPC15 and mated dikaryon dkN001 were studied in submerged liquid cultures containing wood, glucose, and wood plus glucose as carbon sources. The study revealed that this white-rot basidiomycete attacks all the components of the plant cell wall. *P. ostreatus* secretes a variety of glycoside hydrolases, carbohydrate esterases, and polysaccharide lyases, especially when wood is the only carbon source. The presence of wood increased the secretome complexity, whereas glucose diminished the secretion of enzymes involved in cellulose, hemicellulose and pectin degradation. In contrast, the presence of glucose did not influence the secretion of redox enzymes or proteases, which shows the specificity of glucose on the secretion of cellulolytic enzymes. The comparison of the secretomes of monokaryons and dikaryons reveals that secretome complexity is unrelated to the nuclear composition of the strain.

## Introduction

Lignocellulose is a major reservoir of organic carbon on Earth and can be a source of starting molecules for the production of biofuels (cellulose) and platform compounds for biorefinery or synthesis of new chemicals (hemicelluloses and lignin). The understanding of the biological mechanisms involved in the cycling of lignocellulose in nature is of paramount importance in the design of industrial processes for using this bioresource.

Lignin synthesis is a key step in the adaptation of plants from aquatic to terrestrial environments. Traqueophytes are land plants that have lignified tissues (the xylem) for conducting water and minerals throughout the plant, enabling efficient transport and larger sizes of plants. Lignin waterproofs the otherwise permeable (because of hydrophilic polysaccharides) xylem cell walls and provides a structural rigidity that is crucial for plants to reach heights that provide vascular plants a competitive advantage for obtaining sunlight^1^. Furthermore, due to its molecular architecture, lignin is a recalcitrant polymer, where different phenylpropanoid units form a complex three‐dimensional network that is linked by a variety of ether and carbon–carbon bonds^2^ that create a protective layer against pathogens and predators.

Lignin is the second major sink for carbon in plants (after cellulose). It represents as much as 30% of the organic carbon produced in the biosphere^1^ and is the major precursor of coal^3^. In addition to some bacteria and few Ascomycetes (*Xylaria* and *Hypoxylon*), the main lignin degrader organisms are the white rot Agaricomycetes, which have played a key role in carbon recycling of lignocellulose since the onset of lignified compounds in the Carboniferous period^3,4^. Wood-inhabiting Agaricomycetes can secrete a vast diversity of enzymes and are capable of adapting to nearly all terrestrial ecosystems. There are two different methods for degrading lignocellulose: white rot fungi extensively degrade lignin ahead of degrading cellulose, whereas brown rot fungi cause less lignin alteration while principally degrading cellulose. Moreover, some intermediate species share characteristics of both rot types^5^. *Pleurotus ostreatus* is a white rot fungus that degrades lignin to a higher extent than other white rot fungi^6^*.*

The set of proteins secreted by a cell or an organism at a given time is defined as the secretome^7^. These proteins can be set free into the medium, stay attached to the membrane or cell wall, and be integral membrane proteins. Among other functions, fungi use secreted enzymes as a tool to obtain nutrients from their environment, and consequently, protein secretion is a crucial biological process for fungi. The secretome is dependent on the ecological niche of the fungus^8^ and varies following the external conditions to improve fungal growth.

Lignocellulose is composed of three primary compounds: lignin, cellulose and hemicellulose. The wood-decay machinery of the white-rot fungus *Pleurotus ostreatus* targets all these components of the plant cell wall. Many of the secreted enzymes are classified in the CAZy database^9^, and new enzymes involved in this process are still being discovered, such as the auxiliary proteins AA9, AA11, AA14^10^ and AA16^11^ classified as a lytic polysaccharide mono-oxygenase (LPMO) because their activity on polysaccharides has been shown to occur by a novel oxidative mechanism^12^. Furthermore, as found in *P. ostreatus*, many of the genes transcriptionally upregulated during lignocellulose degradation belong to proteins with no known function^13^.

The strategy of basidiomycetes for degrading lignocellulose has another level of complexity: the enzymes involved in this process can be members of multigenic families with a gene-specific regulation^14^. As an example, the genome of the white rot basidiomycete *P. ostreatus* encodes 21 glycoside hydrolase family 5 (GH5, cellulase and other enzymatic activities) genes, while the brown rot basidiomycete *Postia placenta* encodes 12 and the white rot *Phanerochaete chrysosporium* encodes 19 GH5 genes^3^. Furthermore, fungi can regulate the expression and secretion of these enzymes in a temporal sequence that corresponds with the phase of degradation of lignocellulose. The brown rot fungus *P. placenta* degrades lignin in two steps: first, reactive oxygen species are created to attack lignified compounds, and second, enzymes are secreted to hydrolyze polysaccharides from cellulosic compounds^15^. On the other hand, more significant ligninolytic variability exists between white rots regarding enzymes expressed in time and amounts^16^. The expression of a gene involves several processes including transcription, translation, and turnover of messenger RNAs and proteins, and transcriptomics, proteomics, and enzyme activities data are not always related^17,18^. Thus, proteome analysis is a crucial step in verifying the presence of these enzymes acting on their complex lignocellulosic substrates.

Lignocellulose is abundant and renewable, two necessary characteristics of a candidate source of liquid fuels^19^. However, the highly ordered structure of the crystalline cellulose core makes the hydrolysis of this molecule by chemical or biological processes a challenge. The hydrophobic face of cellulose sheets makes cellulose resistant to acid hydrolysis, and the interchain hydrogen bonds make cellulose recalcitrant to enzymatic hydrolysis^20^. Hemicelluloses and lignin create a complex matrix, and lignin is highly recalcitrant, as mentioned above. There are currently more than 1,500 available fungal genomes sequenced, 475 of which come from basidiomycetes (JGI Mycocosm, February 2020)^21^. Among these fungi, white rot basidiomycetes are regarded as an excellent, environmentally friendly alternative to the thermochemical treatments used to overcome the recalcitrance of plant biomass, even with the high cost of this alternative enzymatic treatment^22^. A deeper understanding of the mechanism of lignocellulose degradation by fungi is necessary to reduce costs and make lignocellulosic ethanol a realistic alternative for liquid fuels. *P. ostreatus*, the oyster mushroom, stands out as the third most cultivated edible mushroom and a preferential lignin degrader^6^. These features make this fungus interesting for potential biofuel production and motivate analysis of which enzymes *P. ostreatus* secretes while using lignocellulose as a carbon source.

Basidiomycetes such as *P. ostreatus* offer an unparalleled possibility for studying the genetic control of central processes of their biology. The reproductively competent and fruit-body producing mycelium is a strict dikaryon in which each cell contains two compatible nuclei. By protoplasting a dikaryotic strain it is possible to recover each of the two nuclei in a different monokaryotic strain enabling, by this way, to study the contribution of each one to the genetic control of a process both as monokaryon (i.e., each one of the nuclei in a given cytoplasm) and as dikaryons. This type of study also permits to elucidate some aspects of the dikaryotic lifestyle in comparison with the diploid condition of other eukaryotes.

*Pleurotus ostreatus* dkN001 is a commercial strain able to produce a large number of mushrooms with excellent organoleptic properties. The two nuclei present in it were separated and two monokaryotic strains (called protoclones mkPC9 and mkPC15) were produced^22^ which, upon mating, reconstitute the dkN001. The genomes of mkPC15 and mkPC9 have been sequenced and extensively studied^5,13,23^. The genome sequence offers us the opportunity to predict encoded proteins that can be secreted (bioinformatics secretome^13^) before performing more costly and complex secretome analyses. However, the correlation between bioinformatics (complete set of predicted secreted proteins present in a genome) and the in vitro secretome (actual group of enzymes used in a precise step of lignocellulose degradation, under given culture conditions, by specific sets of cells, and/or at a given time) needs to be established by using proteomics secretome analyses. The two monokaryotic strains behave differently between them and to the dikaryon in submerged cultures, and the transcription of the genes coding for proteins targeted for secretion was also different depending on the nuclear condition and the type of culture^12^.

In this study, we analysed the secretomes of *P. ostreatus* grown in submerged shaking cultures using poplar (*Populus alba*) chips (wood, W), wood supplemented with glucose (WG), or glucose (G) as carbon sources using LC–MS/MS to identify the proteins that *P. ostreatus* uses to adapt to these media. This is the first in vitro secretome analysis comparing *P. ostreatus* sequenced monokaryotic protoclones mkPC15 and mkPC9 which each contain one of the two nuclei present in the dikaryon dkN001^5,23,24^.

The purposes of these experiments were to shed light on the regulation of protein secretion by wood and glucose (end product of cellulose biodegradation), and to determine the effect of the monokaryotic and dikaryotic condition of the fungus on the secretome profile. We considered G cultures as a basal condition, the proteins detected in W cultures were assumed to be induced by wood, and the proteins recovered in WG cultures were considered to be induced by wood but insensitive to glucose repression.

## Results and discussion

### General qualitative analysis of the secretome

In this work, we analysed the proteins recovered from supernatants (secretomes) of the monokaryotic strains mkPC9 and mkPC15 and the dikaryon dkN001 (which harbours the nuclei present in mkPC9 and mkPC15) carried out in 14-days old shaking cultures performed in synthetic medium containing either wood (W), glucose (G), or wood and glucose (WG), as carbon source. Under these experimental conditions, the glucose was not exhausted after 14 days of culture (Supplementary Table S1). 790 proteins that showed at least two positively matched peptides and 1% FDR were identified in the nine secretomes (three strains, three culture conditions, three replicates per each of the nine secretomes, obtained from the culture supernatants. This number included the finding of the same protein in different culture conditions. If we discard these repetitions, the number of different proteins detected was 432. Furthermore, this number included the proteins produced by alleles of the same gene. The allelic proteins were defined as those whose genes met the allelic criterion of sharing an 80% similarity over 80% of the protein sequence as determined by reciprocal BLAST. 154 allelic protein pairs were found in the dkN001 samples (77 allelic pairs detected), and proteins were shared between monokaryons and the dikaryon (88 of mkPC9 and 94 of mkPC15, respectively) (shown in blue in Supplementary Table S2). If the alleles of the same protein were counted only once, 278 different proteins were identified in the nine secretomes studied (Supplementary Table S2).

**Figure 1:**
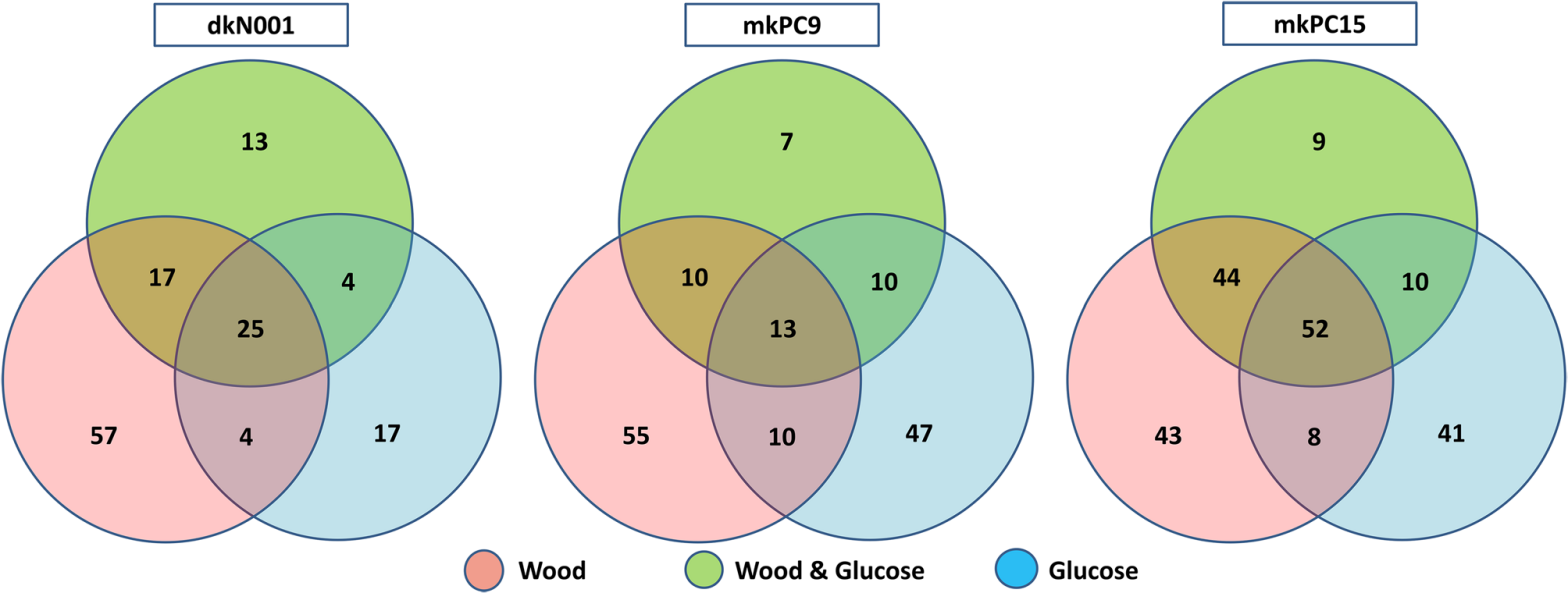
Venn diagram showing the number of proteins identified in the three strains (dkN001, mkPC9 and mkPC15) cultivated using wood (W), sugar (G) or wood and sugar (WG) as a carbon source. The numbers inside the diagram indicate the number of unique proteins identified in each strain and condition.

Figure 1 shows the Venn diagrams of the number of proteins identified per strain and the culture conditions. A total of 136 different proteins were recovered from the dkN001 culture supernatants, 152 from mkPC9 and 207 from mkPC15. This result suggests that the dikaryotic condition is not associated with the secretion of a larger number of different proteins and that protein secretion is not an additive genetic trait.

If we assume that the number of different proteins identified in each secretome reflects its complexity, the results indicate that secretome complexity was higher in W than in G in the three strains (103 vs. 50 different proteins in dkN001 in W and G, respectively; 88 vs. 80 in mkPC9 and 147 vs. 111 in mkPC15). These results suggest that W is an inducer of secretome complexity in *P. ostreatus*. Moreover, the presence of glucose in WG cultures was associated with a decrease in secretome complexity in the three strains (58, 40 and 115 different proteins identified in dkN001, mkPC9 and mkPC15, respectively). This result suggests that G could act as a repressor of global secretome complexity in W cultures. Parenti et al.^25^ have shown the inductor effect of water polluted with wheat straw extracts on *P. ostreatus* laccase production and activity, in concordance with the results reported here. Furthermore, knock-out and overexpression studies of the transcriptional regulator CRE1 for carbon catabolite repression in mkPC9^26^ concur with the effect of glucose on the complexity observed.

In a previous study, Alfaro et al.^13^ showed that the number of genes coding for proteins bioinformatically predicted to be secreted (bioinfosecretome) was similar in mkPC9 and mkPC15 (538 and 554, respectively), that more than 90% of these genes were transcribed and that the bioinfosecretome transcriptional landscape of mkPC9 and mkPC15 was similar in static but not in shaking cultures. In this study performed using shaking cultures, the secretome complexity of mkPC9 and mkPC15 was notably different (88 vs. 147 different proteins identified in mkPC9 vs. mkPC15 in W, 80 vs. 111 in G and 40 vs. 115 in WG) in concordance with previously published results. Surprisingly, however, the slow-growing strain mkPC15 showed a more complex secretome than the fast-growing strains mkPC9 and dkN001.

Wood was the carbon source for which a larger number of specific proteins were secreted (57, 55 and 43 in dkN001, mkPC9 and mkPC15, respectively). In contrast, the number of carbon source-specific proteins detected in G cultures was lower, especially in dkN001. If we consider the number of carbon-source specific proteins as an indicator of the ability to adapt to the carbon source, these results suggest that the monokaryotic strains have similar adaptive ability for both carbon sources, whereas the dikaryotic strain is more adapted to the complex W carbon source than to the basal G.

The higher secretome complexity observed in the slow-growing strain mkPC15 was associated with a reduction in the specificity of its secretome. The carbon source-specific proteins detected in dkN001 and mkPC9 represented 64 and 72% of the proteins identified in these strains, respectively. In PC15, however, the number of carbon source-specific proteins comprised only 45% of the proteins identified in this strain.

### Protein families found in the secretomes

The proteins identified in the LC–MS/MS experiments were individually annotated using different databases (JGI, Interpro, Pfam and Gene Ontology) and manually classified into nine functional groups: glycosyl hydrolases (GH), proteins with unknown function, redox enzymes, intracellular proteins, proteases, esterases/lipases, nonenzyme proteins, lyases, other enzymes (Supplementary Table S2).

**Figure 2:**
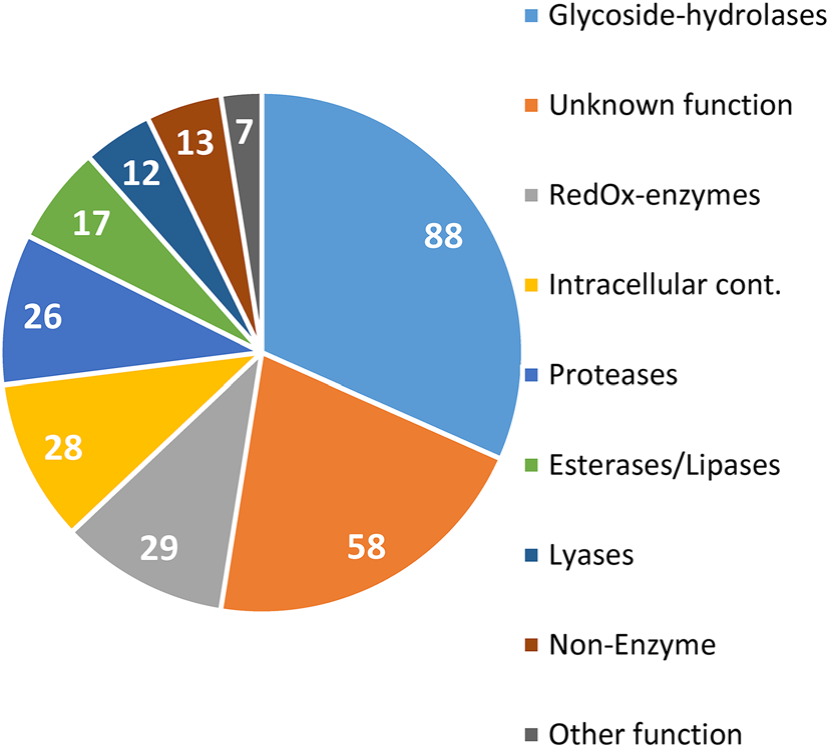
Functional classification of the proteins identified in all samples.

Figure 2 shows that GHs, proteins with unknown functions, redox enzymes, intracellular proteins, and proteases were the most represented groups and accounted for more than 75% of the proteins identified in the secretome. Excluding the group of intracellular proteins that represent only a minimal amount of the bioinfosecretome^13^, the four major protein groups corresponded to the larger groups of secretable proteins identified in the bioinfosecretome. The abundance of intracellular proteins in the experimental secretome could have been caused by hyphae and cell breaks produced while manipulating the mycelium. In addition, it should be kept in mind that only the proteins freely secreted into the culture media were analysed in this study, whereas other secreted proteins that remained attached to the cell wall or the membrane were discarded. Similarly, the abundance of proteins without a known function was higher in the bioinfosecretome and its transcriptome studied in different culture conditions^13^ than in the experimental secretome presented here. This difference can occur because the small molecular size of many of these unknown proteins makes it more difficult to detect them with the LC–MS/MS strategy used here.

**Figure 3:**
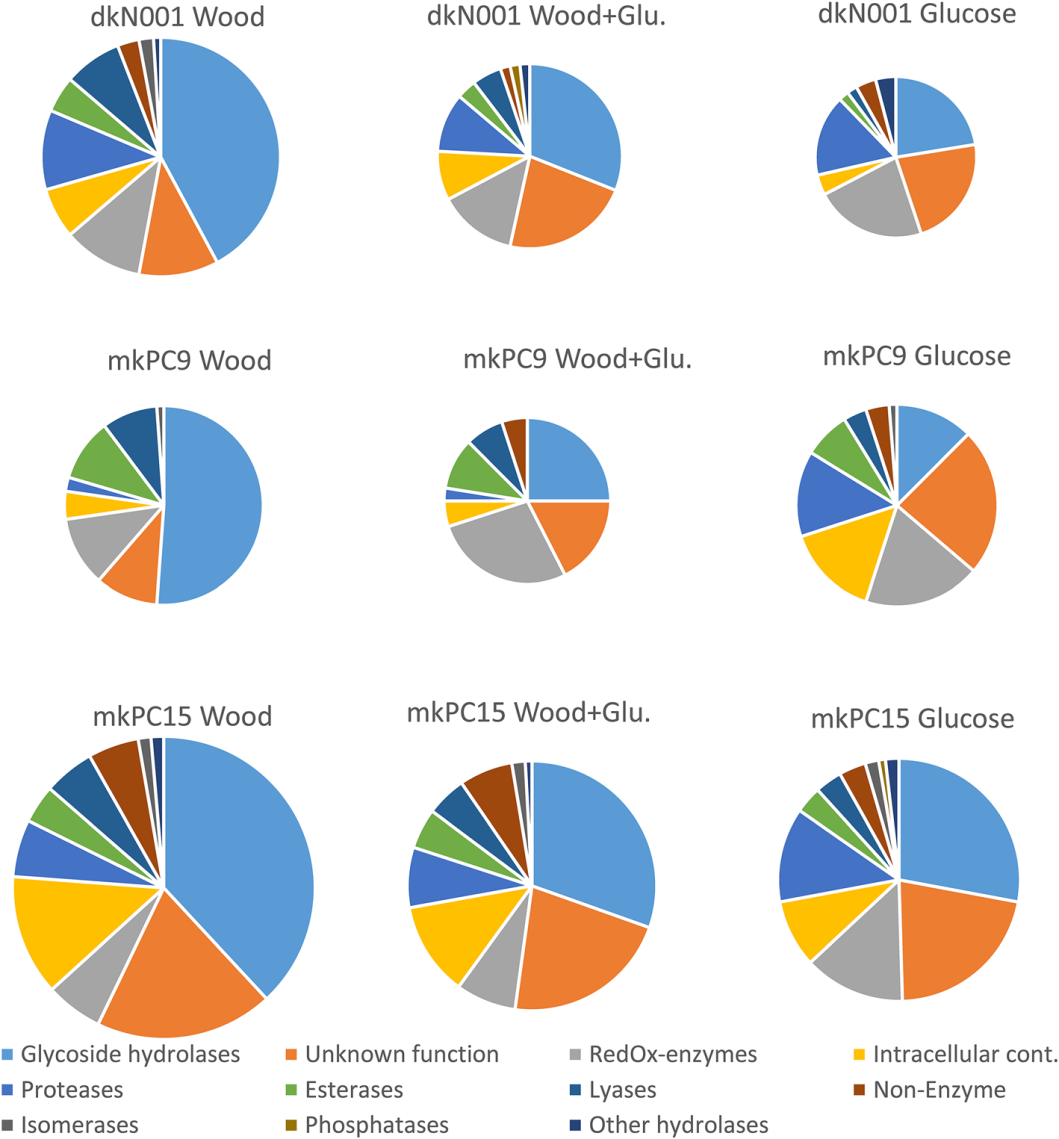
Functional classification of proteins identified in the secretomes of dikaryon N001 and monokaryons PC9 and PC15 cultivated in liquid shaken media containing glucose (G), wood (W) or glucose plus wood (WG) as carbon source. Sizes are proportional to the number of proteins identified.

Figure 3 shows the functional classification of the secreted proteins recovered from the supernatants of the three strains (mkPC15, mkPC9 and dkN001) growing on liquid media containing lignocellulose (wood) or glucose as carbon sources. Taking into account all the proteins identified in the three strains, GHs were the largest group of proteins found in the W cultures, whereas the abundance of these enzymes was lower when glucose was present in the cultures (G and WG). This difference suggests that the presence of glucose in the culture media negatively affects GH secretion in *P. ostreatus*. This behaviour is enzyme group specific as it differs from that of the redox enzymes, whose presence was similar in the three strains and conditions, and from that of the group of proteins with unknown function, whose presence did not seem to be affected by the presence of glucose.

The mkPC15 strain showed a secreted protein profile different to mkPC9 or dkN001 (Fig. 3). In this strain the complexity of the GH secretome is less affected by the presence of Glucose (see data in Supplementary Table S3) than it is in the mkPC9 or dkN001 strains. The number of proteins with unknown function was also higher in this strain than in mkPC9 and dkN001. In solid-state wood degradation experiments, dkN001 and mkPC9 grew on wheat straw equally fast, but mkPC15 was not able to colonise this type of lignocellulose. Compared with the EN113 test (European standard EN 113, 1996) on beech sap wood, dkN001 and mkPC9 degraded 19% and 12% of the wood dry weight biomass, respectively, whereas mkPC15 did not colonise the wood blocks and showed degradation < 1% (data not shown). These results suggest that low wood degradation capacity of mkPC15 could be due to its poorer growing ability and not its lower wood-degrading enzyme secretion. In this context, the higher number of intracellular proteins recovered in the W mkPC15 supernatants in comparison with those recovered in mkPC9 and dkN001 could be an additional symptom of the weaker condition of this strain when growing using complex substrates. These results suggest different abilities to degrade plant biomass-related substrates, as previously described in other fungi, pointing out the importance of strain selection for biotechnological applications^27^.

### Focus on the main secreted protein families

#### Glycoside hydrolases

Eighty-eight different secreted GHs were identified that could be further classified into 35 different CAZy families. GH7 (putative reducing-end cellobiohydrolases, 12 proteins), GH5 (endoglucanases, seven proteins), AA9 (copper-dependent lytic polysaccharide monooxygenases, LPMO, seven proteins), GH28 (polygalacturonases, five proteins), GH16 (endoglucanases and other enzymatic activities^28^, four proteins), and GH43 (arabinofuranosidases, four proteins) were the most represented (Supplementary Table S2). The GH7 and AA9 enzymes are among the largest GH families found in the genome of *P. ostreatus* (16 and 29 gene models, respectively, in the mkPC15 genome). Furthermore, the AA9 LPMO gene family was highly expressed in transcriptome analyses of this strain carried out in other different culture conditions^13^.

Cellulose degradation requires the combined action of the three GH groups that have been found in the secretomes analysed here: endoglucanases (seven GH5, two GH12 and one GH45), cellobiohydrolases (two GH6 and 12 GH7) and β-glucosidases (three GH3) (Supplementary Table S2). GH5 endoglucanases have different enzymatic activities, including β-glucosidase and endoglucanase^9^. These proteins were recovered in dkN001 cultures only when W was the carbon source. In the two monokaryons, however, some members of this family were also found in the G cultures, which can be associated with the broad activity range of these enzymes (see below). LPMOs (AA9) are proposed to open access points in crystalline cellulose to allow cellulases to massively degrade the macromolecule^29^. Seven LPMO proteins were identified only in the W-containing cultures, suggesting that these enzymes are W induced and G repressed (Supplementary Table S2). All the GH7 cellobiohydrolases recovered were also exclusively found in W-containing cultures (W and WG) and were absent in the G cultures, and their abundance was drastically reduced in the WG cultures. Moreover, GH7 enzymes were especially predominant in dkN001 (five out of the top ten proteins detected proteins in W cultures) and were also among the more abundantly secreted proteins in the mkPC9 and mkPC15 W cultures. All these data support that the production of GH7 enzymes is enforced by wood and partially repressed by glucose. In contrast to the preceding families, GH3 β-glucosidases (three different proteins) were only recovered from mkPC15 cultures using G as the carbon source. Although the number of GH3 proteins identified is low, it is interesting that one of the enzymatic activities suggested for the GH5 proteins was β-glucosidase, and this enzyme was recovered from the G cultures as it occurs with the members of the GH3 family. In summary, the secretion of enzymes involved in the initial cellulose degradation seems to be stimulated by the presence of W and partially repressed by the presence of G. In contrast, β-glucosidase secretion seems to behave independently.

Hemicelluloses are complex polymers formed by xylans, glucuronoxylans, arabinoxylans, glucomannans and xyloglucans, as well as several monosaccharides, which necessitates a broad hemicellulolytic portfolio of enzymes in the fungi. Twenty-seven GH hemicellulases were identified in this work and classified into ten CAZy families (GH5, GH10, GH11, GH27, GH30, GH31, GH35, GH44, GH74 and GH115, Supplementary Table S2). In accordance with the secretion of cellulolytic enzymes, hemicellulases were found primarily in the W cultures. Most hemicellulases (12 out of 19) were found only in the W and/or WG cultures but not in the G cultures, which suggests that the production of most of these enzymes was promoted by wood and partially repressed by glucose (Supplementary Table S3). Carbohydrate esterases (CE), especially acetyl-xylan esterases, are also involved in the degradation of hemicelluloses. Nine CE proteins were recovered that could be classified into five families (CE1, CE4, CE12, CE15 and CE16). As in the preceding examples, eight CEs were only recovered from the cultures containing W, and only one was recovered from the cultures containing G as the sole carbon source. As in the case of cellulases, the data reported here suggest that the secretion of hemicellulases is induced by W and partially repressed by G.

A different behaviour, however, was observed in the case of α-galactosidases (GH27, cleaving galactose residues linked to mannan) and β-galactosidases (GH35, cleaving d-galactose residues in some hemicelluloses). Both enzyme families were recovered from all the cultures independently of the carbon source. Interestingly, enzymes of the GH27 family also show α-*N*-acetylgalactosaminidase activity in *Aspergillus niger,* suggesting that members of this family could be involved in processes other than hemicellulose degradation^30,31^.

In plant primary cell walls, the matrix in which the cellulose network is embedded is composed of pectin, a highly hydrated network of polysaccharides rich in galacturonic acid. Polysaccharide lyases (PLs) are the main enzymes that degrade these components. The *P. ostreatus* secretome contained PL1, 3, 4, and 8 family enzymes, which are mainly pectate and rhamnogalacturonan lyases. As in the cases of cellulases and hemicellulases, PL were found more predominantly in the W cultures, although the WG cultures showed a lower abundance (Supplementary Table S2). PL1 and PL3 (pectate lyases) and PL4 (rhamnogalacturonase) were identified as the most abundantly secreted proteins in the W cultures. GH16, 28, 62, 78, 79, 88 and 105 and CE 8, 12 are also involved in pectin degradation and were identified in *P. ostreatus* secretomes.

#### Proteins without a known function

The second largest group of proteins identified in the secretome was formed by proteins without a known function. Fifty-eight proteins were classified in this section, including 24 for which the two alleles were recovered. The genes coding for proteins with unknown function represent 37% of the bioinfosecretome of *P. ostreatus*, and they account for more than 50% of the transcription of the genes coding for proteins targeted for secretion in other reference culture conditions^13^. In the secretomes studied here, the unknown proteins accounted for 21% of the total proteins recovered. These proteins did not show a carbon source-dependent pattern (Supplementary Table S3), and for most of them, the two alleles were recovered from dkN001. No dikaryotic-specific proteins were expressed in dkN001 but not in mkPC9 and/or mkPC15. However, 34 proteins (58.6% of this protein group) were found solely in the monokaryons but not in dkN001. Moreover, 23 of the monokaryotic-specific proteins were only recovered from mkPC15. This value represents 11% of the mkPC15 secretome identified in this study.

#### Redox enzymes

Twenty-nine different redox proteins were recovered from all the cultures independently of the carbon source, although the number of redox enzymes secreted was slightly higher in the G and WG media for the three strains (Fig. 3, Supplementary Table S3). No differences were observed in the secretion patterns of the monokaryons and the dikaryon for these enzymes. The secreted redox proteins could be further classified into different families from which AA5, AA3 and cupredoxins are more represented in the secretome. Copper radical oxidases (AA5) were found in high concentrations in nearly all the media analysed here. Glyoxal oxidase, a peroxide-producing enzyme, is the most intensively studied representative of this CAZy family^32^. Together with glucose-methanol-choline aryl-alcohol oxidases (included in AA3 family), these enzymes may supply extracellular hydrogen peroxide for the fungal wood decay that is carried out by class-II peroxidases^32^. AA5 enzymes are very common enzymes in fungal cultures and have recently been reported to exhibit a broad substrate range^33^, which explains the ubiquity of this CAZy family in the *P. ostreatus* secretomes. Cupredoxin domain-containing proteins were also found in all the strains and culture conditions. Cupredoxins are involved in intermolecular electron transfer reactions and are present in laccases and other multicopper oxidases, which contain three of these domains in their sequence^34^.

Several enzymes belonging to this RedOx classification, such as those belonging to the AA1 and AA2 CAZy classes, are known to have the ability to degrade or modify lignin. The AA1 enzymes are multicopper oxidases including laccases, ferroxidases and multicopper oxidases. Family AA2 contains class II lignin-modifying peroxidases, including manganese peroxidases, versatile peroxidases and lignin peroxidases.

The detection of laccases (AA1_1) deserves special mention since these proteins have been extensively studied in white rot fungi. Only two laccases out of 12 genes in the genomes, Lacc2 (POXA3) and Lacc10 (POXC), were identified in these analyses (Supplementary Table S2). Lacc2 was recovered from the W and WG dkN001 and mkPC9 cultures. Lacc10 was only recovered from dkN001 and mkPC9 containing W as the sole carbon source. No laccases were detected in the mkPC15 cultures. Castanera et al.^14^ and Alfaro et al.^13^ found that Lacc2 and Lacc10 were the main sources of laccase activity in the shaking cultures induced with wheat straw extracts. Fernández-Fueyo et al.^35^ also identified Lacc10 as the most abundant in the secretome of mkPC9 growing on wheat straw or poplar chips. In other systems, such as the *Phlebia radiata*^36^ secretome, laccases were found regardless of the carbon source, suggesting the importance of laccase in other functions ranging from mushroom development to fungus/host interactions and stress defence, in addition to lignin degradation^37^.

Cellobiose dehydrogenase CDH (AA8/AA3_1) is a key enzyme in the recently discovered pathway of cellulose degradation by LPMO^29^. It binds to the cellulose surface despite the absence of a CBM and oxidizes cellobiose^38^. The only gene present in *P. ostreatus* genome has a signal peptide for secretion and corresponding peptides have been found only in wood containing media (W), which is in agreement with their function.

The two alleles of the unique gene in the *P. ostreatus* genome coding for a soluble quinoprotein glucose/sorbosone dehydrogenase have been identified in the *P. ostreatus* secretomes. A new CAZy family (AA12) was created after the discovery of the oxidation activity toward monosaccharides of these proteins^39^. Like CDH, AA12 enzymes contain a cytochrome domain that could transfer electrons to an LPMO^29^ to allow cellulose degradation, and also have a CBM1 domain that could be related with their binding affinity for insoluble cellulose suggesting a role similar to CDH in the extracellular oxidative degradation of cellulose^39^.

All together, these findings highlight the connections between polysaccharide and lignin biodegradation in plant materials. Products from lignin degradation can act as electron donors for cellulose degrading LPMOs, linking the whole lignocellulose fungal degrading process.

#### Proteases

Twenty-six different proteases were identified in the secretomes (Supplementary Table S2), including serine peptidases S8, S9, S10, S28, S33, and S41 and metallopeptidases M28, M35, M36, and M43. The strains dkN001 and mkPC15 secreted more proteases than mkPC9 (Fig. 3). The number of secreted proteases seemed to respond differently to the carbon source in the studied monokaryons (higher in G cultures) than in the dikaryon (higher in W) (Supplementary Table S3). Proteases have a role in fungal cell wall reorganisation and degradation during hyphal growth to recycle essential nutrients^36^. The main function of these enzymes could be nitrogen acquisition from decomposing organic matter proteins in the nitrogen-limited wood environment^40^. Secreted proteases can also have a role in modulating the amount of fungal enzymes in extracellular media, as suggested in *P. ostreatus*^41^ and *P. radiata*^36^.

#### Other proteins

The secretome analyses recovered five cerato-platanin-like proteins. One of these proteins was present in the three strains and the three culture conditions. Cerato-platanins are small secreted defence proteins whose function is not fully understood. Some authors have related them to expansins that were also detected in our secretome analysis. Alfaro et al.^13^ also found that cerato-platanins were actively transcribed in shaking cultures, especially by strain mkPC15. Here, we found that dkN001 is also a strong cerato-platanin producer.

### Conclusions

The secretome of the white-rot fungus *P. ostreatu*s in shaking cultures has revealed that the secretion of enzymes involved in cellulose and hemicellulose degradation is induced by wood and repressed by glucose. This is a selective regulation mechanism since other enzymatic systems such as redox enzymes (including laccases) and proteases were not influenced by the carbon source. Moreover, the secretome complexity was higher in the presence of wood than in the presence of glucose, and this complexity seems to not be associated with the dikaryotic condition but rather with the genetic characteristics of each strain.

## Material and methods

### Fungal strains and culture conditions

Cultures of *P. ostreatus* strains dkN001 (dikaryon), mkPC9 and mkPC15 (monokaryons) were maintained on a malt agar medium (20 g/l malt extract and 15 g/l agar) at 24 °C in the dark. *Populus alba* wood was chopped into particles of approximately 1–5 × 0.5 × 1 mm and dried for three days at 80 °C (moisture content of 4.4% and water content of 4.2%).

Three 10 mm diameter pieces of one-week old agar cultures were used for inoculation of 100 ml liquid pre-cultures containing SMY-media (sucrose 10 g/l, malt extract 10 g/l, yeast extract 4 g/l). After five days of growth in the conditions described below, the mycelium was washed once with sterile water using a nylon filter and resuspended in 200 ml of sterile water. The mycelium was homogenized for 15 s at 8,000 rpm, (Ultraturrax T25, Janke & Kunkel, IKA Labortechnik, Staufen, Germany) and 3 ml aliquots were used as inoculum for the 150 ml experimental cultures. All experiments were prepared in liquid media containing 0.1 g/l Na_2_B_4_O_7_·H_2_O, 0.07 g/l ZnSO_4_·7H_2_O, 0.01 g/l CuSO_4_·5H_2_O, 0.01 g/l MnSO_4_·4H_2_O, 0.01 g/l FeSO_4_·4H_2_O, and 0.01 g/l, (NH_4_)_6_Mo_7_O_2_·4H_2_O. The culture medium was supplemented with glucose (4 g/l) and/or wood (4 g/l), as required for each experiment. All cultures were incubated for 14 days at 24 °C in the dark while shaking (130 rpm).

### Determination of protein concentration

The total protein concentration was measured by the Coomassie Plus assay using a ready-to-use Bradford reagent from Pierce (Thermo Scientific, Bonn, Germany). Ultrapure BSA (GERBU Biochemicals, Gaiberg, Germany) was used as a calibration standard. Fresh samples from fungal culture supernatants were centrifuged for 10 min at 13,000*g* before processing. Concentrated protein samples dissolved in electrophoresis buffers were diluted with pure water to fit the measurement range and to reduce the concentrations of buffer components below the interfering limits for the protein assay.

### Protein extraction

Culture supernatants containing extracellular proteins were separated from mycelia by filtration through Whatman filter paper No. 1 and frozen at − 20 °C overnight. After thawing the samples, insoluble polysaccharides were removed by centrifugation at 13,000*g* for 60 min at 4 °C, and the supernatant was frozen again. The processed supernatants from eight parallel cultures were combined and the proteins were concentrated by freeze-drying.

Freeze-dried samples were dissolved in 100 ml distilled water and centrifuged at 4,000*g* for 30 min. After removing the undissolved debris, SDS, sodium chloride, and sodium deoxycholate were added to the samples to final concentrations of 1% (w/v), 1 M and 0.05% (w/v), respectively^42^. The dissolved proteins were precipitated by addition of trichloroacetic acid (TCA) and phosphotungstic acid^43^. In the first step, TCA was added from a 100% TCA stock solution that contained 100 g TCA in 45.4 ml water to obtain a final TCA concentration of 10%. After mixing, the samples were placed on ice for 30 min. Thereafter, phosphotungstic acid was added from a 10% (w/v) stock solution in water to the final concentration of 0.5%. The samples were mixed well and kept on ice overnight. The precipitated proteins were collected by centrifugation at 25,000*g* for 30 min and precipitation agents were removed through subsequent washings with ice cold 20% Tris-buffer (50 mM, pH 7.5) in acetone (vol/vol). In most instances, three washing steps were required to remove TCA from the protein pellets^44^. Finally, protein samples were washed with pure, ice-cold acetone, air-dried and then stored at − 20 °C for further processing. The recovered proteins were dissolved in 100 mM ammonium bicarbonate and the total protein amount was determined using Coomassie Reagent (Pierce, Germany).

### Protein digestion

Proteins were digested with trypsin in two steps as previously described^45^. Briefly, aliquots containing approximately 500 μg of protein from each experiment (three replicates) dissolved in 100 mM ammonium bicarbonate, were digested with sequencing grade trypsin (Promega, Mannheim, Germany) using an enzyme to substrate ratio of 1:40 (w/w) at 37 °C for 16 h. Thereafter, proteins in the samples were reduced using 1,4-dl-dithiothreitol (DTT) [5 mM) and tris-(2-carboxyethyl)phosphine] (TCEP, 5 mM), and alkylated with iodoacetamide (15 mM). After addition of a new amount of trypsin (enzyme to substrate ratio of 1:50), the samples were digested again for 60 min at 58 °C^46^. The digested peptides were de-salted with a C18 Sep-Pak column (Waters, Milford MA) and dried in a vacuum centrifuge. The samples were dissolved in 20 mM ammonium formate (pH 10) and the total peptide amount was determined using BCA Protein Reagent (Pierce, Germany) calibrated with a tryptic bovine serum albumin (BSA) digest.

### Shotgun protein identification by LC–MS/MS

The digested peptides were first fractionated at a pH of 10^47^ using a Reprosil Gold 3 µm C18 column (150 × 2 mm; Dr. Maisch GmbH, Ammerbuch, Germany). Samples with 300 µg of peptides in 300 µl ammonium formate were separated using a linear gradient of acetonitrile in 20 mM ammonium formate (pH 10). 25 fractions were collected, dried in a vacuum centrifuge and stored at − 20 °C.

Peptide analysis by LC–MS/MS was performed using 1100 HPLC (Agilent, Böblingen, Germany) interfaced to an Esquire 3000 ion trap mass spectrometer (Bruker-Daltonic, Bremen, Germany) via an electrospray ionization (ESI) unit. Each one of the 25 collected peptide fractions was dissolved in 10 μl of 5% (vol/vol) formic acid and 4 μl of each sample was loaded onto a 180 μm i.d. capillary column packed with 3 μm Reprosil-Pur C18-AQ (Dr. Maisch GmbH, Ammerbuch, Germany), conditioned with 98% of solvent A (0.1% formic acid in water) and 2% of solvent B (0.1% formic acid in 90% acetonitrile–water). After 20 min isocratic elution at 2 μl/min, the peptides were eluted using a step-gradient of solvent B: 15% in 5 min, 40% in 90 min, 50% in 5 min, and 90% in 5 min.

The mass spectrometer was set up to take four averages of the MS-spectra (200–1,500 mu) and four averages of the MS/MS-spectra (200–3,000 mu) of the two most abundant precursor ions. The Dynamic Exclusion was set to non-single charged precursor ions and an exclusion time of 1 min. The MS/MS spectra were extracted by DataAnalysis (V. 3.0. Bruker Daltonic, Bremen, Germany) and the peptides were identified using Mascot (V. 2.4, Matrix Science, London, UK). The target database was constructed from annotated genomes of *P. ostreatus* mkPC15 and mkPC9 (https://genome.jgi.doe.gov/PleosPC15_2, https://genome.jgi.doe.gov/PleosPC9_1) and the SwissProt database. Peptide identifications were adjusted to 1% false discovery rate (FDR) against a decoy database. All searches were run as a tryptic digest with one missing cleavage allowed, and a fixed carbamidomethylation of cysteine and variable oxidation of methionine. Mass tolerances were set to 1.4 Da and 0.4 Da for the MS and MS/MS spectra, respectively. Mascot results were extracted from raw DAT-files and transferred to an SQL-database (Microsoft SQL Server 2005). SQL queries were used to extract proteins with at least two peptides with scores higher than the corresponding identity score. In Supplementary Table S2, MUDPit score is the protein score given by MASCOT Program; the sum of the ions scores of all the non-duplicate peptides over the homology threshold. Where there are duplicate peptides, the highest scoring peptide is used. emPAI values were calculated with the MASCOT program which used an emPAI algorithm^48^. In SignalP column we annotate the presence or absence of a signal peptide for secretion using the SignalP program^49^.

### Glucose consumption measurement

We measure the glucose consumption during the 14 days of fungal cultivation using the 3,5-dinitrosalicylic acid (DNSA) method. Three biological replicates of each one of the 9 different medium were taken on days 0, 3, 6, 9, 12 and 14^50^.

## Supplementary information

Supplementary Information

## Data Availability

The datasets generated during and/or analyzed during the current study are available from the corresponding author on reasonable request.

## References

[1] Boerjan W, Ralph J, Baucher M (2003). Lignin biosynthesis. Annu. Rev. Plant Biol..

[2] Ruiz-Dueñas FJ, Martínez AT (2009). Microbial degradation of lignin: How a bulky recalcitrant polymer is efficiently recycled in nature and how we can take advantage of this. Microb. Biotechnol..

[3] Floudas D (2012). The Paleozoic origin of enzymatic lignin decomposition reconstructed from 31 fungal genomes. Science (80-).

[4] Nelsen MP, DiMichele WA, Peters SE, Boyce CK (2016). Delayed fungal evolution did not cause the Paleozoic peak in coal production. Proc. Natl. Acad. Sci. U. S. A..

[5] Riley R (2014). Extensive sampling of basidiomycete genomes demonstrates inadequacy of the white-rot/brown-rot paradigm for wood decay fungi. Proc. Natl. Acad. Sci. U. S. A..

[6] Martínez AT (1994). Progress in biopulping of non-woody materials: Chemical, enzymatic and ultrastructural aspects of wheat straw delignification with ligninolytic fungi from the genus Pleurotus. FEMS Microbiol. Rev..

[7] Tjalsma H, Bolhuis A, Jongbloed JD, Bron S, van Dijl JM (2000). Signal peptide-dependent protein transport in *Bacillus subtilis*: A genome-based survey of the secretome. Microbiol. Mol. Biol. Rev..

[8] Alfaro M, Oguiza JA, Ramírez L, Pisabarro AG (2014). Comparative analysis of secretomes in basidiomycete fungi. J. Proteom..

[9] Lombard V, Golaconda Ramulu H, Drula E, Coutinho PM, Henrissat B (2014). The carbohydrate-active enzymes database (CAZy) in 2013. Nucleic Acids Res..

[10] Couturier M (2018). Lytic xylan oxidases from wood-decay fungi unlock biomass degradation. Nat. Chem. Biol..

[11] Filiatrault-Chastel C (2019). AA16, a new lytic polysaccharide monooxygenase family identified in fungal secretomes. Biotechnol. Biofuels.

[12] Hemsworth GR, Henrissat B, Davies GJ, Walton PH (2014). Discovery and characterization of a new family of lytic polysaccharide monooxygenases. Nat. Chem. Biol..

[13] Alfaro M (2016). Comparative and transcriptional analysis of the predicted secretome in the lignocellulose-degrading basidiomycete fungus *Pleurotus ostreatus*. Environ. Microbiol..

[14] Castanera R (2012). Transcriptional and enzymatic profiling of *Pleurotus ostreatus* laccase genes in submerged and solid-state fermentation cultures. Appl. Environ. Microbiol..

[15] Zhang J (2016). Localizing gene regulation reveals a staggered wood decay mechanism for the brown rot fungus *Postia placenta*. Proc. Natl. Acad. Sci. U. S. A..

[16] Presley GN, Panisko E, Purvine SO, Schilling JS (2018). Coupling secretomics with enzyme activities to compare the temporal process of wood metabolism among white and brown rot fungi. Appl. Environ. Microbiol..

[17] Miyauchi S (2017). The integrative omics of white-rot fungus *Pycnoporus coccineus* reveals co-regulated CAZymes for orchestrated lignocellulose breakdown. PLoS One.

[18] Schwanhäusser B (2011). Global quantification of mammalian gene expression control. Nature.

[19] Rubin EM (2008). Genomics of cellulosic biofuels. Nature.

[20] Himmel ME (2007). Biomass recalcitrance: Engineering plants and enzymes for biofuels production. Science (80-).

[21] Grigoriev IV (2014). MycoCosm portal: Gearing up for 1000 fungal genomes. Nucleic Acids Res..

[22] Hatakka AI (1983). Pretreatment of wheat straw by white-rot fungi for enzymic saccharification of cellulose. Eur. J. Appl. Microbiol. Biotechnol..

[23] Larraya LM (1999). Molecular Karyotype of the White Rot Fungus *Pleurotus ostreatus*. Appl. Environ. Microbiol..

[24] Alfaro M (2017). Genomic, Transcriptomic and Proteomic Analysis of *Pleurotus ostreatus* Secreted Proteins.

[25] Parenti A (2013). Induction of laccase activity in the white rot fungus *Pleurotus ostreatus* using water polluted with wheat straw extracts. Bioresour. Technol..

[26] Yoav S (2018). Effects of cre1 modification in the white-rot fungus *Pleurotus ostreatus* PC9: Altering substrate preference during biological pretreatment. Biotechnol. Biofuels.

[27] López SC (2017). Functional diversity in *Dichomitus squalens* monokaryons. IMA Fungus.

[28] Viborg AH (2019). A subfamily roadmap of the evolutionarily diverse glycoside hydrolase family 16 (GH16). J. Biol. Chem..

[29] Kracher D (2016). Extracellular electron transfer systems fuel cellulose oxidative degradation. Science (80-).

[30] Kulik N (2010). The α-galactosidase type A gene aglA from *Aspergillus niger* encodes a fully functional α-*N*-acetylgalactosaminidase. Glycobiology.

[31] van den Brink J, de Vries RP (2011). Fungal enzyme sets for plant polysaccharide degradation. Appl. Microbiol. Biotechnol..

[32] Kersten P, Cullen D (2014). Copper radical oxidases and related extracellular oxidoreductases of wood-decay Agaricomycetes. Fungal Genet. Biol..

[33] Yin D (2015). Structure–function characterization reveals new catalytic diversity in the galactose oxidase and glyoxal oxidase family. Nat. Commun..

[34] Hoegger PJ, Kilaru S, James TY, Thacker JR, Kües U (2006). Phylogenetic comparison and classification of laccase and related multicopper oxidase protein sequences. FEBS J..

[35] Fernández-Fueyo E (2016). A secretomic view of woody and nonwoody lignocellulose degradation by *Pleurotus ostreatus*. Biotechnol. Biofuels.

[36] Kuuskeri J (2016). Time-scale dynamics of proteome and transcriptome of the white-rot fungus *Phlebia radiata*: Growth on spruce wood and decay effect on lignocellulose. Biotechnol. Biofuels.

[37] Kües U, Rühl M (2011). Multiple multi-copper oxidase gene families in basidiomycetes—what for?. Curr. Genom..

[38] Tan T-C (2015). Structural basis for cellobiose dehydrogenase action during oxidative cellulose degradation. Nat. Commun..

[39] Matsumura H (2014). Discovery of a eukaryotic pyrroloquinoline quinone-dependent oxidoreductase belonging to a new auxiliary activity family in the database of carbohydrate-active enzymes. PLoS One.

[40] Wymelenberg AV (2005). The *Phanerochaete chrysosporium* secretome: Database predictions and initial mass spectrometry peptide identifications in cellulose-grown medium. J. Biotechnol..

[41] Palmieri G, Giardina P, Bianco C, Fontanella B, Sannia G (2000). Copper induction of laccase isoenzymes in the ligninolytic fungus *Pleurotus ostreatus*. Appl. Environ. Microbiol..

[42] Bensadoun A, Weinstein D (1976). Assay of proteins in the presence of interfering materials. Anal. Biochem..

[43] YeetYeang H, Yusof F, Abdullah L (1998). Protein purification for the lowry assay: Acid precipitation of proteins in the presence of sodium dodecyl sulfate and other biological detergents. Anal. Biochem..

[44] Fragner D, Zomorrodi M, Kües U, Majcherczyk A (2009). Optimized protocol for the 2-DE of extracellular proteins from higher basidiomycetes inhabiting lignocellulose. Electrophoresis.

[45] Eastwood DC (2011). The plant cell wall-decomposing machinery underlies the functional diversity of forest fungi. Science (80-).

[46] Havlis J, Thomas H, Sebela M, Shevchenko A (2003). Fast-response proteomics by accelerated in-gel digestion of proteins. Anal. Chem..

[47] Gilar M, Olivova P, Daly AE, Gebler JC (2005). Two-dimensional separation of peptides using RP-RP-HPLC system with different pH in first and second separation dimensions. J. Sep. Sci..

[48] Ishihama Y (2005). Exponentially modified protein abundance index (emPAI) for estimation of absolute protein amount in proteomics by the number of sequenced peptides per protein. Mol. Cell Proteom..

[49] Bendtsen JD, Nielsen H, von Heijne G, Brunak S (2004). Improved prediction of signal peptides: Signa*l*P 3.0. J. Mol. Biol..

[50] Miller GL (1959). Use of dinitrosalicylic acid reagent for determination of reducing sugar. Anal. Chem..

