# Supplementary material for: Glucose counteracts wood-dependent induction of lignocellulolytic enzyme secretion in monokaryon and dikaryon submerged cultures of the white-rot basidiomycete Pleurotus ostreatus
Source: Sci Rep. 2020 Jul 24;10:12421. doi: 10.1038/s41598-020-68969-1 (PMC7381666; doi:10.1038/s41598-020-68969-1)
Supplement: Supplementary file 1 — Supplementary Information [file 41598_2020_68969_MOESM1_ESM.pdf]

Glucose counteracts wood-dependent induction of lignocellulolytic enzyme secretion in monokaryon and dikaryon submerged cultures of the white-rot basidiomycete *Pleurotus ostreatus*

Manuel Alfaro, Andrzej Majcherczyk, Ursula Kües, Lucía Ramírez and Antonio G. Pisabarro*

*Corresponding author, e-mail: gpisabarro@unavarra.es

Supplementary Table 1. Time-course measurement of glucose consumption during 14 days of cultivation of the three fungal strains growing on the different culture media.

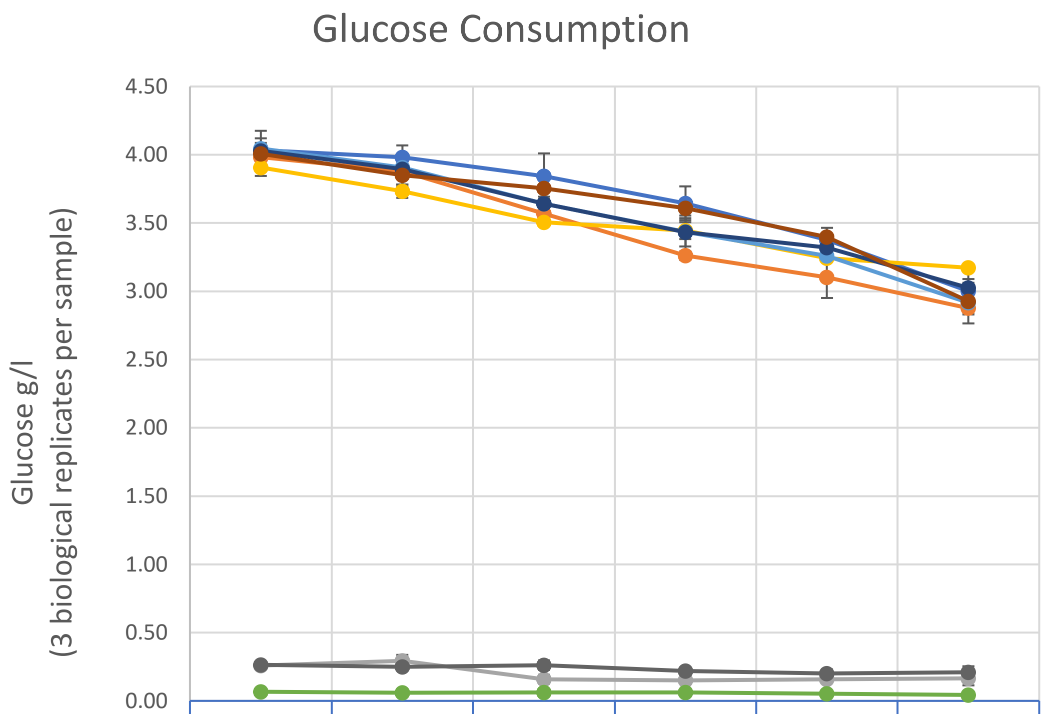

	Day 0	Day 3	Day 6	Day 9	Day 12	Day 14
dkN001G	4.03	3.98	3.84	3.64	3.38	3.00
dkN001WG	3.98	3.88	3.57	3.26	3.10	2.88
dkN001W	0.26	0.29	0.16	0.15	0.16	0.17
mkPc9G	3.91	3.73	3.51	3.44	3.24	3.17
mkPc9WG	4.05	3.91	3.64	3.44	3.26	2.92
mkPc9W	0.07	0.06	0.06	0.06	0.05	0.04
mkPc15G	4.03	3.90	3.64	3.43	3.32	3.03
mkPc15WG	4.01	3.85	3.75	3.61	3.40	2.93
mkPc15W	0.26	0.25	0.26	0.22	0.20	0.21

Supplementary Table 2. List of proteins identified in the 9 different media. Each protein has a MUDpit score(a) or an emPai value(b) if it is present in this media. Allelic pairs are colored in blue.

278 unique proteins				a. MUDpit									b. emPAI										
proteinaccession	ShortName	Allelic pair	Annotation	dkN001			mkPC9			mkPC15			dkN001			mkPC9			mkPC15			SignalP	prot_mass
				W	WG	G	W	WG	G	W	WG	G	W	WG	G	W	WG	G	W	WG	G		
PoPC9FM82810	Esterases/Lipases	1	Carbohydrate esterase (put.), CE16						526									0.74				Q (0.143/ 25)	35583
PoPC15FM1075485	Esterases/Lipases	2	Carbohydrate esterase CE16	1335									1.53									S (0.994/ 17)	17677
PoPC9FM96445	Esterases/Lipases	2	Carbohydrate esterase CE16	1942			823						1.72			1.12						S (0.994/ 17)	33685
PoPC15FM1097054	Esterases/Lipases	3	Carbohydrate esterase, CE1		235									0.38								S (1.000/ 22)	34742
PoPC9FM112290	Esterases/Lipases	3	Carbohydrate esterase, CE1		164									0								S (1.000/ 22)	34377
PoPC15FM1114413	Esterases/Lipases	4	Carbohydrate esterase, CE1, acetylxylan esterase A	959						455	965		0									S (0.992/ 25)	39351
PoPC9FM117351	Esterases/Lipases	4	Carbohydrate esterase, CE1, acetylxylan esterase A	959									0.77									S (0.980/ 25)	39351
PoPC15FM1102068	Esterases/Lipases	5	Carbohydrate esterase, CE12 (put. rhamnogalacturonan acetylesterase)							290									0.69			S (0.998/ 20)	26636
PoPC15FM1086797	Esterases/Lipases	6	Carbohydrate esterase, CE15 (put. Glucuronoyl esterase)	150							599		0.2									S (1.000/ 20)	46835
PoPC9FM89668	Esterases/Lipases	6	Carbohydrate esterase, CE15 (put. Glucuronoyl esterase)	150			148						0			0.28						Q (0.073/ 19)	33902
PoPC15FM1078467	Esterases/Lipases	7	Carbohydrate esterase, CE4 (polysaccharide deacetylase)	176									0.66									S (1.000/ 20)	27458
PoPC9FM125794	Esterases/Lipases	7	Carbohydrate esterase, CE4 (polysaccharide deacetylase)				279									0.66						S (1.000/ 20)	27398
PoPC15FM1111329	Esterases/Lipases	8	Carbohydrate esterase, CE4 (put. chitin deacetylase)		418					581	864	344		0								S (0.997/ 19)	48694
PoPC9FM115623	Esterases/Lipases	8	Carbohydrate esterase, CE4 (put. chitin deacetylase)		418		410	519	963					0.12		0.12	0.19	0.42				S (0.997/ 19)	48680
PoPC15FM175606	Esterases/Lipases	9	Carbohydrate esterase, CE4 (put. chitin deacetylase)								124											S (0.999/ 20)	49740
PoPC15FM1044335	Esterases/Lipases	10	Carbohydrate esterase, CE8 (put. pectin methylesterase)									642									0.7	S (0.965/ 18)	42576
PoPC15FM1061918	Esterases/Lipases	11	Carbohydrate esterase, CE8, pectin methylesterase							978	456	822								0.74		S (1.000/ 23)	35686
PoPC9FM116926	Esterases/Lipases	11	Carbohydrate esterase, CE8, pectin methylesterase				2138									1.2						S (1.000/ 23)	35735
PoPC15FM1091241	Esterases/Lipases	12	Carboxylesterase			759						589			0							S (0.999/ 20)	59574
PoPC9FM75940	Esterases/Lipases	12	Carboxylesterase			759			1237						0.46			0.95				S (0.999/ 20)	59588
PoPC9FM114288	Esterases/Lipases	13	Carboxylesterase (type B)						334									0.33				S (0.999/ 20)	59432
PoPC9FM116339	Esterases/Lipases	14	Carboxylesterase, type B (put. carotenoid ester lipase)				1888	201	666							1.14	0.27	0.53				S (0.992/ 19)	59763
PoPC9FM126566	Esterases/Lipases	15	Carboxylesterase, type B (put. carotenoid ester lipase)				2000	249	588							1.36	0.4	0				S (0.992/ 19)	59452
PoPC15FM1113799	Esterases/Lipases	16	Esterase (put. rhamnogalacturan acetylesterase)							556									1.19			S (0.711/ 23)	24783
PoPC9FM78619	Esterases/Lipases	16	Esterase (put. rhamnogalacturan acetylesterase)				375									0.39						S (0.951/ 19)	25435
PoPC15FM1078000	Esterases/Lipases	17	GDSL-like lipase/acylhydrolase (extracellular?)	300						858	619		0						0.64	0.45		Q (0.006/ 38)	45574
PoPC9FM97290	Esterases/Lipases	17	GDSL-like lipase/acylhydrolase (extracellular?)	300			687	267					0.38			0.67	0.29					Q (0.022/ 28)	44105
PoPC15FM1062416	Glycosidases	18	GH72 1,3-beta-glucanosyltransferase, GH72; Glycolipid anchored surface protein GAS1	967	3254	1628				1369	2088	3679	0	0.47	0.27				0.4	0.7	0.54	S (0.983/ 25)	59089
PoPC9FM115581	Glycosidases	18	GH72 1,3-beta-glucanosyltransferase, GH72; Glycolipid anchored surface protein (GAS1)	980	3254	1628	1466	1324	1146				0.4	0	0	0	0.54	0				S (0.983/ 25)	59139
PoPC15FM162095	Glycosidases	19	GH115Alpha-glucuronidase, GH115	2207	150					1618	369		0.87	0								S (1.000/ 22)	109727
PoPC9FM127463	Glycosidases	19	GH115 Alpha-glucuronidase, GH115	2207	150		2235						0	0.05		1.56						S (1.000/ 22)	109749
PoPC15FM176747	Glycosidases	20	GH43 Alpha-N-arabinofuranosidase, GH43	348						1053	142		0									S (0.998/ 20)	34049
PoPC9FM97623	Glycosidases	20	GH43 Alpha-N-arabinofuranosidase, GH43	348									0.18									S (0.998/ 20)	34109
PoPC15FM1094009	Glycosidases	21	GH20 Beta-N-acetylhexosaminidase-like, GH20									2319									1.47	S (1.000/ 20)	59855
PoPC15FM48333	Glycosidases	22	GH6 cellulose 1,4-β-cellobiosidase (non-reducing end), GH6	661						659	772	140	0									S (1.000/ 21)	47673
PoPC9FM45206	Glycosidases	22	GH6 cellulose 1,4-β-cellobiosidase (non-reducing end), GH6	661			707						0.35			0.35						S (1.000/ 21)	47705
PoPC15FM27620	Glycosidases	23	GH7 Endo-1,4-beta-D-glucan-cellobiohydrolase (endo-cellulase) GH7	2675	457					661	253		1.91	0.31					0.8	0.38		S (1.000/ 19)	53203
PoPC9FM83320	Glycosidases	23	GH7 Endo-1,4-beta-D-glucan-cellobiohydrolase (endo-cellulose) GH7	2921	461		3252	1200					1.91	0.31		1.48	1.23					S (1.000/ 19)	53217
PoPC15FM1038048	Glycosidases	24	GH7 Endo-1,4-beta-D-glucan-cellobiohydrolase (endo-cellulose) GH7	765	161					2500	1864		0.67	0								S (1.000/ 19)	49855
PoPC9FM47406	Glycosidases	24	GH7 Endo-1,4-beta-D-glucan-cellobiohydrolase (endo-cellulose) GH7	765	161		958						0	0		0						S (1.000/ 19)	49825
PoPC15FM1039666	Glycosidases	25	GH7 Endo-1,4-beta-D-glucan-cellobiohydrolase (endo-cellulose) GH7	920	246								0	0								S (1.000/ 19)	53998
PoPC9FM49445	Glycosidases	25	GH7 Endo-1,4-beta-D-glucan-cellobiohydrolase (endo-cellulose) GH7	920	246		249						0	0		0						S (1.000/ 19)	53998
PoPC15FM1039504	Glycosidases	26	GH7 Endo-1,4-beta-D-glucan-cellobiohydrolase (endo-cellulose) GH7	4062	488					217	335		4.12	0.76					0.48	0.57		S (0.999/ 19)	50319
PoPC9FM83849	Glycosidases	26	GH7 Endo-1,4-beta-D-glucan-cellobiohydrolase (endo-cellulose) GH7	4028	481		1286						4.12	0		1.2						S (0.999/ 19)	50287
PoPC15FM1092970	Glycosidases	27	GH7 Endo-1,4-beta-D-glucan-cellobiohydrolase (endo-cellulose) GH7	4334	1746					1358	602		0	0					1	0.82		S (1.000/ 19)	57122
PoPC9FM114771	Glycosidases	27	GH7 Endo-1,4-beta-D-glucan-cellobiohydrolase (endo-cellulose) GH7	4334	1746		1852	597					2.47	1.33		0	0					S (1.000/ 19)	57094
PoPC15FM1079521	Glycosidases	28	GH11 Endo-1,4-beta-xylanase GH11	3867						6273	3408		0.71						1.12	1.12		S (0.999/ 20)	25842
PoPC9FM89740	Glycosidases	28	GH11 Endo-1,4-beta-xylanase GH11	3867									0									S (0.999/ 20)	24285
PoPC15FM1098194	Glycosidases	29	GH11 Xylanase GH11	4056						7606	3806		0									S (0.997/ 20)	30603
PoPC9FM110996	Glycosidases	29	GH11 Endo-1,4-beta-xylanase GH11	4056			1642						0.73			0.73						S (0.997/ 20)	30587
PoPC15FM1091692	Glycosidases	30	GH10 Endo-1,4-beta-xylanase, GH10	786						493	1483		0									S (1.000/ 20)	33914
PoPC9FM81650	Glycosidases	30	GH10 Endo-1,4-beta-xylanase, GH10	786									1.41									S (1.000/ 20)	38401
PoPC15FM1047798	Glycosidases	31	GH10 Endo-1,4-beta-xylanase, GH10	1335						1816	261		0									S (1.000/ 20)	43071
PoPC9FM125911	Glycosidases	31	GH10 Endo-1,4-beta-xylanase, GH10	1335			183						0.69			0.3						S (1.000/ 20)	43225
PoPC15FM1078540	Glycosidases	32	GH10 Endo-1,4-beta-xylanase, GH10	547						546	705	756	0									S (1.000/ 19)	35984
PoPC9FM96691	Glycosidases	32	GH10 Endo-1,4-beta-xylanase, GH10	547			192						0.48			0.37						S (1.000/ 19)	35984
PoPC15FM1061994	Glycosidases	33	GH53 Endo-beta-1,4-galactanase, GH53	958	181					1445	541		0	0.35								S (0.994/ 21)	37864
PoPC9FM116896	Glycosidases	33	GH53 Endo-beta-1,4-galactanase, GH53	958	181		1192	315					0.95	0		0.95	0.35					S (0.995/ 21)	37823
PoPC15FM1035805	Glycosidases	34	GH45 Endoglucanase, GH45 (fragment)	893						403			0									Q (0.032/ 29)	25632
PoPC9FM54135	Glycosidases	34	GH45 Endoglucanase, GH45 (fragment)	893			940	288					0.47			0.47	0.33					Q (0.032/ 29)	29047
PoPC15FM152719	Glycosidases	35	GH62 Glycoside hydrolase, GH 62 (alpha-L-arabinofuranosidase)	375						134			0									S (1.000/ 18)	42477
PoPC9FM121125	Glycosidases	35	GH62 Glycoside hydrolase, GH 62 (alpha-L-arabinofuranosidase)	375									0.14									S (1.000/ 18)	42477
PoPC15FM50765	Glycosidases	36	GH12 Glycoside hydrolase, GH12 (put. endo-beta-1,4-glucanase)	292						513			0									S (0.995/ 18)	26225

proteinaccession	ShortName	Allelic pair	ProteinName	dkN001			mkPC9			mkPC15			dkN001			mkPC9			mkPC15			SignalP	prot_mass
				W	WG	G	W	WG	G	W	WG	G	W	WG	G	W	WG	G	W	WG	G		
PoPC9FM84996	Glycosidases	36	GH12 Glycoside hydrolase, GH12 (put. endo-beta-1,4-glucanase)	292				406					0.7				0.53					S (0.999/ 18)	26222
PoPC15FM1095839	Glycosidases	37	GH13 Glycoside hydrolase, GH13 (alpha-amylase)	319								107	0									S (0.983/ 23)	62232
PoPC9FM65411	Glycosidases	37	GH13 Glycoside hydrolase, GH13 (alpha-amylase)	319			453						0.44			0.32						S (0.986/ 23)	62239
PoPC15FM29106	Glycosidases	38	GH15 Glycoside hydrolase, GH15, Glucoamylase	511						722		414	0									S (1.000/ 19)	61586
PoPC9FM124117	Glycosidases	38	GH15 Glycoside hydrolase, GH15, Glucoamylase	511			926						0.38			0.74						S (1.000/ 19)	61544
PoPC15FM1064585	Glycosidases	39	GH16 Glycoside hydrolase, GH16	177						288		367	0									S (0.984/ 22)	41888
PoPC9FM132563	Glycosidases	39	GH16 Glycoside hydrolase, GH16	177									0.22									S (0.984/ 22)	41936
PoPC15FM40942	Glycosidases	40	GH16 Glycoside hydrolase, GH16 (Beta-glucan synthesis-associated protein (SKN1))							304		296							0.39		0.23	Q (0.000/ 0)	68585
PoPC9FM87194	Glycosidases	40	GH16 Glycoside hydrolase, GH16 (Beta-glucan synthesis-associated protein (SKN1))				101									0.13						Q (0.000/ 0)	68582
PoPC15FM1040267	Glycosidases	41	GH16 Glycoside hydrolase, GH16 (put. Endo-1,3(4)-beta-glucanase) Endo-beta-glucanase GH family 16	360		256							0		0							S (0.996/ 22)	35376
PoPC9FM82945	Glycosidases	41	GH16 Glycoside hydrolase, GH16 (put. Endo-1,3(4)-beta-glucanase)	360		256							0.27		0.17							S (0.997/ 23)	35507
PoPC15FM1076482	Glycosidases	42	GH16 Glycoside hydrolase, GH16 (put. endo-1,3(4)-beta-glucanase)							295		620							0.51		0.51	S (0.999/ 19)	34114
PoPC9FM60047	Glycosidases	43	GH18 Glycoside hydrolase, GH18 (put. chitinase, fragment)		219									0.78								Q (0.243/ 16)	33966
PoPC15FM1035175	Glycosidases	44	GH27 Glycoside hydrolase, GH27 (put. alpha-galactosidase, melibiase) fragment	313	275	212				1208	1735	1218	0.47	0.57	0.21				0.9	1.02	0.67	Q (0.000/ 0)	44028
PoPC9FM90219	Glycosidases	44	GH27 Probable alpha-galactosidase, GH27	240	232		150						0	0		0						Q (0.000/ 33)	57349
PoPC15FM1088219	Glycosidases	45	GH27 Glycoside hydrolase, GH27 (put. alpha-galactosidase, melibiase) fragment							673	937	699							0.6	1.02	0.6	S (0.998/ 20)	48285
PoPC15FM51341	Glycosidases	46	GH27 Glycoside hydrolase, GH27, alpha-galactosidase							873	241								0.69	0.39		S (0.972/ 23)	42999
PoPC9FM90953	Glycosidases	47	GH28 Glycoside hydrolase, GH28				485									0.52						S (0.962/ 20)	47457
PoPC15FM1048073	Glycosidases	48	GH28 Glycoside hydrolase, GH28 (put. (exo)polygalacturonase)							681												S (1.000/ 22)	49063
PoPC9FM59334	Glycosidases	48	GH28 Glycoside hydrolase, GH28 (put. (exo)polygalacturonase)				264									0.34						S (1.000/ 22)	49003
PoPC15FM39721	Glycosidases	49	GH28 Glycoside hydrolase, GH28 (put. exo-polygalacturonase)		179					387	269			0								S (0.998/ 18)	42296
PoPC9FM85018	Glycosidases	49	GH28 Glycoside hydrolase, GH28 (put. exo-polygalacturonase) fragment(?)		179		552	481						0.26		0.48	0.37					Q (0.000/ 45)	35987
PoPC15FM1040639	Glycosidases	50	GH28 Glycoside hydrolase, GH28 (put. Polygalacturonase)							259	452	664										S (1.000/ 17)	36770
PoPC9FM51760	Glycosidases	50	GH28 Glycoside hydrolase, GH28 (put. Polygalacturonase)				2509		185							1.32		0.26				S (1.000/ 17)	36770
PoPC15FM1050633	Glycosidases	51	GH28 Glycoside hydrolase, GH28 (put. Polygalacturonase), fragment		237	355				602	859	1074		0	0							Q (0.000/ 25)	41459
PoPC9FM89478	Glycosidases	51	GH28 Glycoside hydrolase, GH28 (put. Polygalacturonase)		237	355	922							0.21	0.21	0.46						S (0.999/ 24)	44930
PoPC15FM41613	Glycosidases	52	GH3 Glycoside hydrolase, GH3 (put. beta-glucosidase) fragment?									215									0.25	Q (0.001/ 20)	76753
PoPC15FM1035754	Glycosidases	53	GH3 Glycoside hydrolase, GH3 (put. exo-1,4-beta-xylosidase)									326									0.32	S (0.999/ 22)	83373
PoPC15FM1049518	Glycosidases	54	GH3 Glycoside hydrolase, GH3 (put. exo-1,4-beta-xylosidase)									194									0.27	S (0.998/ 18)	84063
PoPC15FM1061735	Glycosidases	55	GH31 Glycoside hydrolase, GH31 (put. alpha-glucosidase)			202						5572			0						1.63	S (1.000/ 20)	106815
PoPC9FM91487	Glycosidases	55	GH31 Glycoside hydrolase, GH31 (put. alpha-glucosidase)			225			255						0.2			0.17				S (1.000/ 22)	107820
PoPC15FM1079718	Glycosidases	56	GH32 Glycoside hydrolase, GH32							336									0.41			Q (0.257/ 21)	58424
PoPC15FM1105441	Glycosidases	57	GH35 Glycoside hydrolase, GH35 (beta-galactosidase)									184									0.11	S (0.999/ 25)	110709
PoPC15FM1066752	Glycosidases	58	GH35 Glycoside hydrolase, GH35 (put. beta-galactosidase)									277									0.13	S (0.860/ 30)	113442
PoPC15FM31763	Glycosidases	59	GH35 Glycoside hydrolase, GH35 (put. beta-galactosidase)									169										S (0.963/ 32)	113175
PoPC15FM1099647	Glycosidases	60	GH43 Glycoside hydrolase, GH43 (put. α-L-arabinofuranoside arabinofuranohydrolase)							641												S (0.990/ 21)	33650
PoPC9FM45547	Glycosidases	60	GH43 Glycoside hydrolase, GH43 (put. α-L-arabinofuranoside arabinofuranohydrolase)				713									0.75						S (0.990/ 21)	30107
PoPC9FM127963	Glycosidases	61	GH43 Glycoside hydrolase, GH43 (put. alpha-L-arabinofuranosidase)				294									0.26						S (0.991/ 23)	36339
PoPC15FM1074766	Glycosidases	62	GH43 Glycoside hydrolase, GH43, (put. galactan 1,3-beta-galactosidase)	637						586	584		0.6						1.15	0.6		S (1.000/ 21)	48207
PoPC9FM54867	Glycosidases	62	GH43 Glycoside hydrolase, GH43, (put. galactan 1,3-beta-galactosidase)	414			796						0			0						S (1.000/ 22)	48092
PoPC15FM1036707	Glycosidases	63	GH44 Glycoside hydrolase, GH44	405									0									S (1.000/ 22)	81934
PoPC9FM54923	Glycosidases	63	GH44 Glycoside hydrolase, GH44	405			922						0.28			0.52						S (1.000/ 22)	81862
PoPC15FM1053206	Glycosidases	64	GH47 Glycoside hydrolase, GH47 (alpha-mannosidase)	1176	643	1744				452	394	1836	0	0	0				0.34	0.27	1.17	S (0.992/ 20)	58769
PoPC9FM88568	Glycosidases	64	GH47 Glycoside hydrolase, GH47 (alpha-mannosidase)	1228	643	1803			1533				0.7	0.21	1.06			0.87				S (0.992/ 20)	58784
PoPC15FM1035282	Glycosidases	65	GH47 Glycoside hydrolase, GH47 (alpha-mannosidase)			130									0							S (0.996/ 22)	58922
PoPC9FM61416	Glycosidases	65	GH47 Glycoside hydrolase, GH47 (alpha-mannosidase)			130			237						0			0				S (0.996/ 22)	58938
PoPC15FM1060958	Glycosidases	66	GH5 Glycoside hydrolase, GH5	262						185	315		0									S (0.994/ 20)	61799
PoPC9FM75659	Glycosidases	66	GH5 Glycoside hydrolase, GH5	262					299				0.26					0.2				S (0.994/ 20)	61799
PoPC15FM42791	Glycosidases	67	GH5 Glycoside hydrolase, GH5							110		175							0.07		0.11	A (0.000/ 19)	80986
PoPC9FM114400	Glycosidases	67	GH5 Glycoside hydrolase, GH5 (put. exo-beta-1,3-glucanase) fragment				139		103							0.07		0.07				A (0.000/ 19)	80955
PoPC15FM1067505	Glycosidases	68	GH5 Glycoside hydrolase, GH5 (endo-beta-1,4-glucanase, cellulase)	340						340			0									S (1.000/ 21)	41225
PoPC9FM116228	Glycosidases	68	GH5 Glycoside hydrolase, GH5 (endo-beta-1,4-glucanase, cellulase)	340									0.41									S (1.000/ 21)	41225
PoPC9FM125269	Glycosidases	69	GH5 Glycoside hydrolase, GH5 (put. endo-beta-1,4-glucanase, cellulase)				291									0.6						S (0.994/ 21)	42128
PoPC15FM1073165	Glycosidases	70	GH6 Glycoside hydrolase, GH6 (cellobiohydrolase)							206												S (0.999/ 23)	46931
PoPC9FM43698	Glycosidases	70	GH6 Glycoside hydrolase, GH6 (cellobiohydrolase)				184									0.35						S (0.999/ 23)	47329
PoPC9FM87701	Glycosidases	71	GH61 Glycoside hydrolase, AA9	73									0.16									S (1.000/ 21)	38891
PoPC15FM1078534	Glycosidases	72	GH61 Glycoside hydrolase, AA9							192												S (1.000/ 20)	29444
PoPC15FM1048891	Glycosidases	73	GH61 Glycoside hydrolase, AA)(cellulase)							383	264											S (0.999/ 20)	22841
PoPC9FM59310	Glycosidases	73	GH61 Glycoside hydrolase, AA9(cellulase)				544									0.44						S (0.999/ 20)	22841
PoPC15FM1032886	Glycosidases	74	GH61 Glycoside hydrolase, AA9(cellulase, endo-1,4-β-D-glucanase), fragment	289									0									S (0.999/ 18)	25898
PoPC9FM46220	Glycosidases	74	GH61 Glycoside hydrolase, AA9 (cellulase, endo-1,4-β-D-glucanase)	289									0.29									S (0.999/ 20)	33305
PoPC15FM1098874	Glycosidases	75	GH61 Glycoside hydrolase, AA9 (cellulase, endoglucanase)							131												S (0.999/ 19)	25917
PoPC15FM1083326	Glycosidases	76	GH61 Glycoside hydrolase, AA9 (endoglucanase)	1063						1097			0									S (1.000/ 18)	24504
PoPC9FM122311	Glycosidases	76	GH61 Glycoside hydrolase, AA9 (endoglucanase)	1063			666						0.95			1.44						S (1.000/ 18)	24847
PoPC15FM18797	Glycosidases	77	GH61 Glycoside hydrolase, AA9 (endoglucanase), fragment							455	276											Q (0.257/ 18)	21589

proteinaccession	ShortName	Allelic pair	ProteinName	dkN001			mkPC9			mkPC15			dkN001			mkPC9			mkPC15			SignalP	prot_mass
				W	WG	G	W	WG	G	W	WG	G	W	WG	G	W	WG	G	W	WG	G		
PoPC9FM90565	Glycosidases	78	GH7 Glycoside hydrolase, GH7 (cbhI-2, cellulose 1,4-beta-cellobiosidase, exoglucanase)				284									0						S (0.999/ 19)	56720
PoPC9FM100398	Glycosidases	79	GH7 Glycoside hydrolase, GH7 (cbhI-3, cellulose 1,4-beta-cellobiosidase, exoglucanase)				1890									1.01						S (1.000/ 19)	56786
PoPC15FM1105963	Glycosidases	80	GH7 Glycoside hydrolase, GH7 (cbhI-3, cellulose 1,4-beta-cellobiosidase, exoglucanase)							266									0.22			S (1.000/ 19)	56846
PoPC9FM107842	Glycosidases	80	GH7 Glycoside hydrolase, GH7 (cbhI-3, cellulose 1,4-beta-cellobiosidase, exoglucanase)				1832									0						S (1.000/ 19)	56786
PoPC9FM129783	Glycosidases	81	GH7 Glycoside hydrolase, GH7 (cbhI-4, cellulose 1,4-beta-cellobiosidase, exoglucanase)				1493									0						S (1.000/ 19)	56804
PoPC15FM1075797	Glycosidases	82	GH7 Glycoside hydrolase, GH7 (Cellulase C)	326						276	560		0									S (0.991/ 19)	54910
PoPC15FM167046	Glycosidases	83	GH7 Glycoside hydrolase, GH7 (Cellulase C)	326						276	560		0									S (0.991/ 19)	55823
PoPC9FM49686	Glycosidases	83	GH7 Glycoside hydrolase, GH7 (Cellulase C)	326			1340						0			0						S (0.991/ 19)	55823
PoPC9FM47295	Glycosidases	84	GH7 Glycoside hydrolase, GH7 (Cellulase C)	326			1340						0.23			0.58						S (0.997/ 19)	55926
PoPC15FM1021086	Glycosidases	85	GH71 Glycoside hydrolase, GH71 (alpha-1,3-glucanase) fragment?	175	288					147	279	382	0.15	0.15						0.23	0.41	Q (0.053/ 22)	41244
PoPC9FM85055	Glycosidases	85	GH71 Glycoside hydrolase, GH71 (alpha-1,3-glucanase) fragment?	175	288								0	0								Q (0.000/ 35)	44869
PoPC15FM1111407	Glycosidases	86	GH74 Glycoside hydrolase, GH74 (put. endoglucanase, cellulase)	515	237								0.3	0.15								Q (0.002/ 28)	99611
PoPC9FM85526	Glycosidases	86	GH74 Glycoside hydrolase, GH74 (put. endoglucanase, cellulase)	466									0									S (1.000/ 22)	84754
PoPC15FM1046326	Glycosidases	87	GH74 Glycoside hydrolase, GH74 (put. xyloglucanase)							227												S (0.999/ 21)	84937
PoPC15FM1064904	Glycosidases	88	GH76 Glycoside hydrolase, GH76 (put. alpha-1,6-mannanase)		103					188	182	173		0.16					0.16	0.44	0.34	S (0.999/ 22)	38748
PoPC9FM115209	Glycosidases	88	GH76 Glycoside hydrolase, GH76 (put. alpha-1,6-mannanase)		103									0								S (0.999/ 22)	38750
PoPC15FM1046178	Glycosidases	89	GH78 Glycoside hydrolase, GH78 (put. alpha-L-rhamnosidase)									637										S (0.999/ 21)	72827
PoPC15FM13903	Glycosidases	90	GH78 Glycoside hydrolase, GH78, alpha-L-rhamnosidase (fragment)							212		250							0.24		0.24	Q (0.002/ 27)	65819
PoPC9FM58710	Glycosidases	90	GH78 Glycoside hydrolase, GH78, alpha-L-rhamnosidase				968									0.61						S (0.991/ 16)	71491
PoPC15FM1038769	Glycosidases	91	GH79 Glycoside hydrolase, GH79	1198						630	285		0									S (0.995/ 29)	49870
PoPC9FM48699	Glycosidases	91	GH79 Glycoside hydrolase, GH79	1198			760	168					0.76			0.33	0.12					S (0.995/ 29)	49930
PoPC9FM116181	Glycosidases	92	GH79 Glycoside hydrolase, GH79			178			248						0.23			0.23				S (0.959/ 22)	55828
PoPC15FM1067626	Glycosidases	93	GH79 Glycoside hydrolase, GH79			120				255	293	1537			0				0.38	0.48	0.68	Q (0.000/ 24)	43509
PoPC9FM116292	Glycosidases	93	GH79 Glycoside hydrolase, GH79			132			178						0.16			0.34				S (0.999/ 19)	58133
PoPC15FM1031712	Glycosidases	94	GH88 Glycoside hydrolase, GH88 (d-4,5-unsaturated β-glucuronyl hydrolase)							122		987									0.67	S (0.996/ 16)	43818
PoPC15FM1040099	Glycosidases	95	GH92 Glycoside hydrolase, GH92 (alpha-1,2-mannosidase)							235									0.14			S (0.993/ 26)	84778
PoPC15FM1042025	Glycosidases	96	GH92 Glycoside hydrolase, GH92 (alpha-1,2-mannosidase)	172	642	195							0	0	0							Q (0.003/ 35)	78707
PoPC9FM84985	Glycosidases	96	GH92 Glycoside hydrolase, GH92 (alpha-1,2-mannosidase)	422	1051	312	145	250	311				0.44	0.7	0.33	0.28	0.23	0.28				Q (0.004/ 29)	69953
PoPC15FM1040697	Glycosidases	97	GH92 Glycoside hydrolase, GH92 (alpha-1,2-mannosidase)									202									0.3	S (0.999/ 23)	86848
PoPC9FM128131	Glycosidases	98	GH105 Glycosyl hydrolase, GH105 (put. unsaturated rhamnogalacturonyl hydrolase)				157									0.37						S (1.000/ 24)	44777
PoPC15FM1041534	Glycosidases	99	GH5 Glycosyl hydrolase, GH5 (endo-1,4-beta-mannosidase)	210									0									S (0.998/ 20)	48326
PoPC9FM123701	Glycosidases	99	GH5 Glycosyl hydrolase, GH5 (endo-1,4-beta-mannosidase)	210									0.27									S (0.998/ 22)	47649
PoPC15FM1041397	Glycosidases	100	GH5 Put. glycoside hydrolase, GH5, fragment								503									0.3		Q (0.000/ 37)	20719
PoPC15FM159035	Glycosidases	101	GH76 Put. Glycoside hydrolase, GH76 (1,6-alpha-mannanase)									585									0.72	S (0.999/ 19)	41813
PoPC15FM1025794	Glycosidases	102	GH5 Putative glycoside hydrolase (GH5), fragment	106						666	754		0									Q (0.349/ 18)	14410
PoPC9FM85079	Glycosidases	102	GH5 Putative glycoside hydrolase (GH5)	347			263	190					0.34			0.26	0.12					S (0.998/ 27)	48401
PoPC15FM1078081	Glycosidases	103	GH131	2119						1385			0									S (0.998/ 20)	31724
PoPC9FM126936	Glycosidases	103	GH131	2119			1445						0.89			0.89						S (0.998/ 20)	35311
PoPC15FM1063776	Glycosidases	104	GH105 Unsaturated rhamnogalacturonyl hydrolase, GH105	494		585				1100	313	485	0.22		0.5				0.83	0.14	0.22	S (0.983/ 18)	41796
PoPC9FM47522	Glycosidases	104	GH105 Unsaturated rhamnogalacturonyl hydrolase, GH105	448	154	611	503						0.22	0.14	0.5	0.31						S (0.983/ 18)	41782
PoPC15FM1108932	Glycosidases	105	GH12 xyloglucan-specific endo-beta-1,4-glucanase, GH12	1752			4507						0.5						0.66	0.5		S (1.000/ 18)	27531
PoPC9FM61738	Glycosidases	105	GH12 Xyloglucan-specific endo-beta-1,4-glucanase, GH12							3602	1051					0						S (1.000/ 18)	29424
PoPC15FM1090291	Intracellular cont.	106	Acyl-CoA binding protein								562											Q (0.000/ 21)	11539
PoPC15FM1049583	Intracellular cont.	107	Amidohydrolase 2	261	594	864					170	2970	0	0	3.58						4.26	Q (0.000/ 41)	19834
PoPC9FM62096	Intracellular cont.	107	Amidohydrolase 2	315	818	1419		469	786				0.87	0.87	1.91		0.43	1.23				Q (0.038/ 21)	31395
PoPC15FM1090792	Intracellular cont.	108	Calmodulin	463						559			0									Q (0.000/ 0)	16814
PoPC9FM73763	Intracellular cont.	108	Calmodulin	463			419						0.92			1.25						Q (0.000/ 0)	16814
PoPC15FM1087719	Intracellular cont.	109	HSP70 protein family							277												Q (0.003/ 23)	71215
PoPC15FM62237	Intracellular cont.	110	Hsp70-like protein	411						801			0									S (0.862/ 38)	73366
PoPC9FM87846	Intracellular cont.	110	Hsp70-like protein	411			335						0.26			0.26						S (0.904/ 38)	73340
PoPC9FM88922	Intracellular cont.	111	Lactonase						268									0.34				Q (0.011/ 45)	38362
PoPC9FM62259	Intracellular cont.	112	Lactonohydrolase-like protein		215				337					0.14				0.31				Q (0.000/ 17)	41917
PoPC15FM1090484	Intracellular cont.	113	LsmAD-domain containing protein							77												Q (0.001/ 73)	88504
PoPC15FM1089714	Intracellular cont.	114	Polyubiquitin							2689	2936	3452										Q (0.000/ 20)	34201
PoPC9FM71298	Intracellular cont.	114	Polyubiquitin						2077									0				Q (0.000/ 20)	23013
PoPC15FM1058300	Intracellular cont.	115	Polyubiquitin							2689	2936	3452										Q (0.000/ 20)	34201
PoPC9FM109117	Intracellular cont.	115	Polyubiquitin						2077									0				Q (0.000/ 21)	21823
PoPC9FM108832	Intracellular cont.	116	Polyubiquitin						2077									0				Q (0.000/ 51)	23749
PoPC15FM1081506	Intracellular cont.	117	Polyubiquitin							2689	2936	3452										Q (0.000/ 20)	24070
PoPC15FM1082432	Intracellular cont.	118	Polyubiquitin							2689	2936	3452										Q (0.000/ 20)	24071
PoPC9FM105181	Intracellular cont.	118	Polyubiquitin						2068									0				Q (0.000/ 20)	22758
PoPC15FM1088454	Intracellular cont.	119	Polyubiquitin							2689	2936	3452										Q (0.000/ 20)	42734
PoPC9FM95693	Intracellular cont.	119	Polyubiquitin						2077									0				Q (0.000/ 20)	33535
PoPC15FM1105321	Intracellular cont.	120	Put. Actin-binding protein							361									0.19			Q (0.000/ 16)	99760
PoPC15FM1095212	Intracellular cont.	121	Put. DNase I-like	178	389								0	0.5								Q (0.001/ 28)	34803
PoPC9FM69654	Intracellular cont.	121	Put. DNase I-like	178	389								0.27	0								Q (0.001/ 28)	34760
PoPC15FM52279	Intracellular cont.	122	Put. formamidase									138										Q (0.000/ 0)	43482
PoPC15FM1041927	Intracellular cont.	123	Put. NAD(P)-reductase							146									0.68			Q (0.057/ 27)	15773
PoPC15FM1103731	Intracellular cont.	124	Putative transcription antitermination protein, NusG	933			710						2.13						2.93	0.98		Q (0.000/ 0)	11642
PoPC9FM83036	Intracellular cont.	124	Putative transcription antitermination protein, NusG	1483	320					1097	169		1.49	0.98		2.13						Q (0.000/ 0)	11614
PoPC15FM1062961	Intracellular cont.	125	Ribonuclease T2							389	230	963							0.77		0.92	S (0.999/ 22)	34501
PoPC9FM110190	Intracellular cont.	125	Ribonuclease T2						435									0.5				S (0.998/ 22)	34452
PoPC15FM1113129	Intracellular cont.	126	Ribosomal protein S27a							2689	2936	3452										Q (0.000/ 20)	18364
PoPC15FM1088775	Intracellular cont.	127	Thioredoxin							714	336											Q (0.000/ 32)	12191
PoPC9FM101837	Intracellular cont.	128	Ubiquitin						2077									0				Q (0.001/ 20)	8649

proteinaccession	ShortName	Allelic pair	ProteinName	dkN001			mkPC9			mkPC15			dkN001			mkPC9			mkPC15			SignalP	prot_mass
				W	WG	G	W	WG	G	W	WG	G	W	WG	G	W	WG	G	W	WG	G		
PoPC15FM1074537	Intracellular cont.	129	Ubiquitin							2689	2936	3452										Q (0.001/ 20)	8649
PoPC9FM66181	Intracellular cont.	129	Ubiquitin						2077									0				Q (0.001/ 20)	8649
PoPC15FM1087569	Intracellular cont.	130	Unknown protein	966	230		1133	1367	315				0.82	0					0.82	0.82		Q (0.000/ 0)	13415
PoPC9FM91272	Intracellular cont.	130	Unknown protein	1320	325	256				504	376		1.2	1.2	1.2	1.2	1.86	1.2				Q (0.000/ 25)	9946
PoPC15FM1046273	Intracellular cont.	131	Unknown protein								174											Q (0.000/ 0)	22651
PoPC15FM1096735	Intracellular cont.	132	Unknown protein							104												Q (0.000/ 28)	44929
PoPC15FM1088793	Intracellular cont.	133	Unknown protein (fragment?)	427						213			2.72						1.2			Q (0.041/ 25)	10018
PoPC15FM35957	Isomerases	134	Aldose-1-epimerase	322						1573	2356	160	0									Q (0.002/ 17)	37268
PoPC9FM113478	Isomerases	134	Aldose 1-epimerase	322			165		1364				0.43			0.24		1.04				S (1.000/ 20)	39519
PoPC15FM1094896	Isomerases	135	Aldose-1-epimerase	322						1573	2356	160	0							0.97		S (1.000/ 20)	41720
PoPC15FM1054118	Lyases	136	Chondroitin AC/alginate lyase , heparinase II/III family like protein	1292	2243	3083				2613	3856	1558	0	0	0				1.17			A (0.000/ 67)	85018
PoPC9FM115859	Lyases	136	Heparinase II/III family protein	1292	2243	3083	2162	1475	751				0.35	0.55	0.71	0.77	0.71	0				A (0.000/ 67)	85032
PoPC15FM1078793	Lyases	137	Oxalate decarboxylase	137	1665							294	0.34	1.26							0.26	S (1.000/ 20)	48586
PoPC9FM55739	Lyases	137	Oxalate decarboxylase	127	1238			1919	1958				0.34	1.26			2.02	2.02				S (1.000/ 20)	48600
PoPC15FM1113870	Lyases	138	Pectate lyase, PL Family 3	3407						6676	641		3.15						6.64	1.5		S (0.999/ 19)	27448
PoPC9FM116196	Lyases	138	Pectate lyase, PL Family 3	3527			3101						2.42			3.65						S (0.999/ 19)	27233
PoPC15FM1045048	Lyases	139	Polysaccharide lyase, PL1 (pectate lyase)	537						368			0									S (1.000/ 19)	33028
PoPC9FM100039	Lyases	139	Polysaccharide lyase, PL1 (pectate lyase)	537			1068						0.4			0.4						S (0.999/ 19)	33076
PoPC15FM1075634	Lyases	140	Polysaccharide lyase, PL1 (put. pectate lyase)	376	152								0.41	0								S (1.000/ 19)	32246
PoPC9FM123331	Lyases	140	Polysaccharide lyase, PL1 (put. pectate lyase)	376	152		812						0	0.42		1.01						S (1.000/ 19)	32232
PoPC15FM1054721	Lyases	141	Polysaccharide lyase, PL1 (put. pectate lyase)	616									0									S (1.000/ 19)	32510
PoPC9FM83989	Lyases	141	Polysaccharide lyase, PL1 (put. pectate lyase)	616			1659						0.41			0.98						S (1.000/ 19)	32697
PoPC15FM199583	Lyases	142	Polysaccharide lyase, PL1 (put. pectate lyase)							584									0.23			S (0.889/ 28)	55829
PoPC15FM1089616	Lyases	143	Polysaccharide lyase, PL14								268	336										S (0.999/ 22)	37978
PoPC15FM1044820	Lyases	144	Polysaccharide lyase, PL4 (rhamnogalacturonase)	486						549	90		0						0.75			S (0.999/ 20)	55583
PoPC9FM58117	Lyases	144	Polysaccharide lyase, PL4 (rhamnogalacturonase)	486			567						0.51			0.67						S (1.000/ 20)	55549
PoPC15FM1111478	Lyases	145	Polysaccharide lyase, PL8							466	330	113							0.42		0.07	S (1.000/ 25)	81382
PoPC9FM53101	Lyases	145	Polysaccharide lyase, PL8				324	305	597							0	0.32	0				S (1.000/ 25)	81472
PoPC15FM1060380	Lyases	146	Put. poly(beta-D-mannuronate) lyase							194												S (0.997/ 30)	52347
PoPC15FM1109346	Lyases	147	Rhamnogalacturonase B PL4	891						2787	1604		0.96						4.54	1.97		S (0.999/ 19)	54684
PoPC9FM128966	Lyases	147	Rhamnogalacturonase B PL4	993			4712						0.96			5.16						S (0.999/ 19)	54643
PoPC15FM1102507	Non-Enzyme	148	Carbohydrate-Binding Module protein, CBM13			311									0							S (1.000/ 21)	17151
PoPC9FM100980	Non-Enzyme	148	Carbohydrate-Binding Module protein, CBM13			311									0.6							S (1.000/ 23)	17335
PoPC15FM1113946	Non-Enzyme	149	Carbohydrate-Binding Module protein, CBM13							165	126											Q (0.000/ 38)	36595
PoPC15FM1089845	Non-Enzyme	150	CBM13 protein (ricin B-like lectin)							393	320								1.91	1.04		Q (0.001/ 20)	15179
PoPC15FM1069722	Non-Enzyme	151	Cerato-platanin like protein	458						898	759	187	0.44						1.48	0.73	0.44	S (0.997/ 21)	14827
PoPC15FM1081025	Non-Enzyme	152	Cerato-platanin like protein	526	1121	136				619	481	1265	0	0	0							S (1.000/ 21)	15071
PoPC9FM102212	Non-Enzyme	152	Cerato-platanin like protein	526	1121	136		323	360				0.71	1.05	0.43		0.71	0.71				S (1.000/ 21)	15071
PoPC9FM45615	Non-Enzyme	153	Cerato-platanin like protein						388									3.42				S (1.000/ 19)	14516
PoPC15FM1088025	Non-Enzyme	154	Cerato-platanin like protein							232												S (0.999/ 21)	14687
PoPC9FM44086	Non-Enzyme	154	Cerato-platanin like protein					96									0.44					S (0.999/ 21)	14793
PoPC15FM1110827	Non-Enzyme	155	Cerato-platanin like protein							356	191	2834							0.42	1.03	2.45	S (0.993/ 21)	15310
PoPC15FM172522	Non-Enzyme	156	Put. expansin family protein							216	2856								0.49	2.05		S (1.000/ 21)	35289
PoPC9FM54071	Non-Enzyme	157	Put. expansin like protein						374									0				S (1.000/ 21)	19255
PoPC15FM1096037	Non-Enzyme	158	Put. expansin like protein							382	596	1262										S (0.999/ 21)	31541
PoPC15FM1096058	Non-Enzyme	159	Put. expansin like protein								417											S (0.994/ 31)	31546
PoPC15FM21503	Non-Enzyme	160	Thaumatin-like protein	111									0									S (0.989/ 20)	27794
PoPC9FM46813	Non-Enzyme	160	Thaumatin-like protein	111									0.35									S (0.989/ 20)	27794
PoPC15FM1112538	Other hydrolases	161	Glyoxalase I (put.)							595												Q (0.000/ 0)	18401
PoPC15FM185836	Other hydrolases	162	Ribonuclease T2			246						630			0							S (0.999/ 17)	41532
PoPC9FM78300	Other hydrolases	162	Ribonuclease T2			246									0.4							S (0.999/ 17)	41663
PoPC15FM157064	Other hydrolases	163	Unknown protein (put. Glutaminase A)	1797	1920	978				1388	632	1767	0.8	0.8	0.44				0.61	0.39	0.74	S (0.999/ 23)	77586
PoPC9FM83417	Other hydrolases	163	Unknown protein (put. Glutaminase A)	1203	1394	795							0	0	0							S (0.999/ 23)	70524
PoPC15FM1110233	Phosphatases	164	Put. histidine acid phosphatase (phytase)		269									0.23								S (0.820/ 19)	41482
PoPC9FM82770	Phosphatases	164	Put. histidine acid phosphatase (phytase), fragment		291									0.37								Q (0.000/ 0)	35835
PoPC15FM1090758	Phosphatases	165	Put. Metallophosphoesterase									205										S (0.995/ 18)	43113
PoPC9FM115072	Proteases	166	Hydrolase family, put. Peptidase S33	205									0.2									S (0.998/ 22)	61437
PoPC15FM166434	Proteases	167	Peptidase family A1 (pepsin A)									537										S (1.000/ 20)	43147
PoPC15FM1089322	Proteases	168	Peptidase family A1 (put. pepsin A)									200										S (0.999/ 18)	60626
PoPC9FM115140	Proteases	168	Peptidase family A1 (put. pepsin A)						426									0.36				S (0.999/ 18)	55055
PoPC15FM1055405	Proteases	169	Peptidase family A1A (aspartic endopeptidase, saccharopepsin/pepsin A)									281										S (0.994/ 17)	43578
PoPC15FM1037634	Proteases	170	Peptidase family M35 (peptidyl-Lys metalloendopeptidase), fragment	316						1132			0						0.78			Q (0.000/ 0)	34163
PoPC9FM52745	Proteases	170	Peptidase family M35 (peptidyl-Lys metalloendopeptidase)	316									0.7									S (0.998/ 24)	37093
PoPC15FM1092788	Proteases	171	Peptidase family M43 (subfamily M43B), sequence problem	163									0.1									S (0.999/ 18)	57851
PoPC9FM67725	Proteases	171	Peptidase family M43 (subfamily M43B)	163									0									S (0.999/ 18)	29997
PoPC15FM1078405	Proteases	172	Peptidase family S10 (serine carboxypeptidase)			321					221	206			0							S (0.999/ 20)	52057
PoPC9FM127085	Proteases	172	Peptidase family S10 (serine carboxypeptidase)			321									0.46							S (0.999/ 20)	52072
PoPC15FM1104326	Proteases	173	Peptidase family S10, (carboxypeptidases)	302	549							416	0	0								S (0.785/ 42)	60680
PoPC9FM51352	Proteases	173	Peptidase family S10, (carboxypeptidases), fragment	302	549								0.43	0.23								Q (0.000/ 0)	55690
PoPC15FM1105014	Proteases	174	Peptidase family S28 (serine peptidase)			246						1384			0							S (0.997/ 24)	62400
PoPC9FM85063	Proteases	174	Peptidase family S28 (serine peptidase)			246			367						0.25			0.31				S (0.997/ 24)	63203
PoPC15FM1102733	Proteases	175	Peptidase family S33	150						1229			0.2						0.72			S (0.973/ 24)	63020
PoPC9FM123302	Proteases	175	Peptidase family S33	150									0									S (0.995/ 24)	58817
PoPC15FM175905	Proteases	176	Peptidase family S33	180									0									S (0.840/ 19)	59920
PoPC9FM84016	Proteases	176	Peptidase family S33	156									0									S (0.817/ 19)	56611
PoPC15FM1111906	Proteases	177	Peptidase family S33 (put. fragment)	405	258	233							0	0	0							Q (0.000/ 30)	59482
PoPC9FM132884	Proteases	177	Peptidase family S33 (put. fragment)	405	258	233							0.27	0.21	0.27							Q (0.000/ 30)	59475
PoPC9FM47034	Proteases	178	Peptidase family S53						201									0.2				S (1.000/ 20)	62955
PoPC15FM1064502	Proteases	179	Peptidase family S53 (put. Kumamolisin)									246									0.18	S (0.999/ 21)	67393
PoPC15FM1087304	Proteases	180	Peptidase family S9							315									0.37			Q (0.001/ 21)	71971
PoPC15FM1079957	Proteases	181	Peptidase M28 (subfamily M28E)									1578										S (0.940/ 17)	48781
PoPC9FM115424	Proteases	182	Peptidase M28 (subfamily M28E)			170									0.51							S (0.978/ 24)	27075
PoPC15FM1113156	Proteases	183	Peptidase M28, aminopeptidase							1579	537								2.29			S (1.000/ 19)	40248
PoPC9FM88371	Proteases	183	Peptidase M28, aminopeptidase						308									0				S (1.000/ 19)	40263

proteinaccession	ShortName	Allelic pair	ProteinName	dkN001			mkPC9			mkPC15			dkN001			mkPC9			mkPC15			SignalP	prot_mass
				W	WG	G	W	WG	G	W	WG	G	W	WG	G	W	WG	G	W	WG	G		
PoPC15FM1059702	Proteases	184	Peptidase M28E, aminopeptidase							861	1342											S (1.000/ 21)	40339
PoPC15FM62198	Proteases	185	Peptidase M36, fungalysin	400	285	383				370	586	1087	0.25	0.25	0.2				0.2	0.31	0.5	S (0.997/ 24)	63101
PoPC9FM91073	Proteases	185	Peptidase M36, fungalysin	439	388	398			703				0.25	0.25	0.2			0.43				S (0.997/ 24)	63101
PoPC15FM175915	Proteases	186	Peptidase S10, serine carboxypeptidase	524	1039					1189	652	1075	0	0							0.98	S (0.943/ 27)	58035
PoPC9FM83972	Proteases	186	Peptidase S10, serine carboxypeptidase	524	1039				199				0.48	0.63				0.55				S (0.976/ 27)	57982
PoPC15FM1066015	Proteases	187	Peptidase S10, serine carboxypeptidase	593	814	308				251	384	1765	0.32	0	0					0.27	0.43	S (0.995/ 21)	72058
PoPC9FM88317	Proteases	187	Peptidase S10, serine carboxypeptidase	593	814	308	379		369				0	0.32	0.22	0		0.27				S (0.982/ 21)	72070
PoPC15FM1077652	Proteases	188	Peptidase S53, propeptide (tripeptidyl-peptidase SED2)	640	502	1791					332	708	0.31	0.2	0.56					0.25	0.31	S (0.999/ 22)	63765
PoPC9FM60171	Proteases	188	Peptidase S53, propeptide (tripeptidyl-peptidase SED2)<br>Peptidase S53, propeptide	400	360	1237	90		1012				0	0	0	0		0				S (0.999/ 22)	63934
PoPC15FM1088548	Proteases	189	Peptidase S8, subtilisin-related protein							512	544	522							0.44	0.34	0.44	S (1.000/ 20)	38731
PoPC9FM71759	Proteases	189	Peptidase S8, subtilisin-related protein					84	2677								0.24	0.92				S (1.000/ 20)	38714
PoPC15FM1045574	Proteases	190	Peptidase family S28 (carboxypeptidase)			135					331	337			0							S (0.986/ 19)	60918
PoPC9FM57949	Proteases	190	Peptidase family S28 (carboxypeptidase)			135			184						0.21			0.26				S (0.986/ 19)	60865
PoPC9FM82641	Proteases	191	Putative peptidase family S41						964									0.68				S (0.997/ 24)	71629
PoPC15FM1064574	RedOx-enzymes	192	Amino acid oxidase (put.)			606				208					0				0.33			Q (0.000/ 0)	70572
PoPC9FM114605	RedOx-enzymes	192	Amino acid oxidase (put.)			606									0.38							Q (0.000/ 0)	70405
PoPC15FM1067653	RedOx-enzymes	193	AA3_2 Aryl-alcohol oxidase	144	691	221							0	0	0							S (1.000/ 23)	63914
PoPC9FM116309	RedOx-enzymes	193	AA3_2 Aryl-alcohol oxidase	144	691	221			1251				0.14	0.43	0.14			0.86				S (1.000/ 23)	63900
PoPC9FM93955	RedOx-enzymes	194	AAO3 Aryl-alcohol oxidase						418									0.37				S (0.985/ 26)	63604
PoPC15FM1087553	RedOx-enzymes	195	AA3_2 Aryl-alcohol oxidase (GMC-oxidase)									656									0.3	S (0.999/ 21)	64445
PoPC9FM69649	RedOx-enzymes	195	AA3_2 Aryl-alcohol oxidase (GMC-oxidase)						844									0				S (1.000/ 21)	62081
PoPC15FM41743	RedOx-enzymes	196	CDH Cellobiose dehydrogenase (acceptor), fragment							337	222											Q (0.025/ 23)	79101
PoPC9FM62103	RedOx-enzymes	196	CDH Cellobiose dehydrogenase (acceptor), fragment				734									0.29						Q (0.025/ 23)	79101
PoPC15FM1081617	RedOx-enzymes	197	AA5_1 Copper radical oxidase	1482	6730	7024							0	1.16	0							S (1.000/ 24)	81453
PoPC9FM101121	RedOx-enzymes	197	AA5_1 Copper radical oxidase	1352	6649	7003	497	502	2119				0.75	1.08	1.08	0	0.47	0.69				S (0.996/ 24)	81760
PoPC15FM1109334	RedOx-enzymes	198	AA5_1 Copper radical oxidase									333									0.14	S (0.996/ 31)	63253
PoPC15FM1114640	RedOx-enzymes	199	AA5_1 Copper radical oxidase	2168	4185	3630							0	0	0.55							S (0.999/ 23)	111932
PoPC9FM134564	RedOx-enzymes	199	AA5_1 Copper radical oxidase	2244	4216	3630	2470	774	2463				0.6	1.02	0	0.68	0.37	0.73				S (0.999/ 23)	109927
PoPC15FM1079389	RedOx-enzymes	200	AA5_1 Copper radical oxidase (put. glyoxal oxidase)	97	520	442						468	0.07	0	0							S (1.000/ 22)	82268
PoPC9FM88952	RedOx-enzymes	200	AA5_1 Copper radical oxidase (put. glyoxal oxidase), fragment	97	520	442		136					0	0.37	0.43		0.13					Q (0.001/ 16)	72254
PoPC15FM1090764	RedOx-enzymes	201	AA5_1 Copper radical oxidase (put. glyoxal oxidase)	2530	9297	5481				2792	7612	6241	0	0	0							S (1.000/ 22)	71025
PoPC9FM62166	RedOx-enzymes	201	AA5_1 Copper radical oxidase (put. glyoxal oxidase)	2530	9297	5481	3856	5588	2605				0.68	1.51	1.22	1.31	1.61	1.22				S (1.000/ 22)	71427
PoPC9FM84350	RedOx-enzymes	202	AA5_1 Copper radical oxidase (put. glyoxal oxidase)					72									0					S (0.956/ 40)	70549
PoPC15FM1097654	RedOx-enzymes	203	Cupredoxin domain containing protein	2117	9299	4609				647	627	426	0	0	0							S (1.000/ 20)	14530
PoPC9FM87572	RedOx-enzymes	203	Cupredoxin domain containing protein	2117	9299	4609	1447	17754	6509				1.66	6.07	7.6	2.93	7.6	6.07				S (1.000/ 20)	13779
PoPC9FM115057	RedOx-enzymes	204	Dye decolorizing peroxidase (DyP2)				621									0.49						S (0.975/ 24)	56572
PoPC15FM62084	RedOx-enzymes	205	FAD-dependent oxidase									4045										S (1.000/ 20)	61910
PoPC9FM100586	RedOx-enzymes	206	FAD-linked oxidase (unknown function)					254	881								0.11	0.57				S (0.996/ 21)	56287
PoPC15FM1101230	RedOx-enzymes	207	AA7 FAD-oxidase (put. glucooligosaccharide oxidase)							376	3803	5471									2.04	S (0.992/ 27)	53578
PoPC9FM90315	RedOx-enzymes	207	AA7 FAD-oxidase (put. glucooligosaccharide oxidase)					226	647								0.45	0.53				S (0.992/ 27)	53551
PoPC15FM1037343	RedOx-enzymes	208	AA3_2 Glucose oxidase (GMC-oxidase, FAD)									268										S (0.997/ 24)	69624
PoPC9FM91123	RedOx-enzymes	208	AA3_2 Glucose oxidase (GMC-oxidase, FAD)						837									0.34				S (0.600/ 24)	68809
PoPC15FM154703	RedOx-enzymes	209	AA3_2 Glucose oxidase (GMC-oxidase, FAD)									373									0.18	S (0.784/ 24)	70053
PoPC9FM130566	RedOx-enzymes	209	AA3_2 Glucose oxidase (GMC-oxidase, FAD)						671									0				S (0.998/ 24)	63414
PoPC15FM1088797	RedOx-enzymes	210	AA3_2 GMC oxidoreductase<br>GMC oxidoreductase							473	274	442										S (0.992/ 21)	72170
PoPC15FM1067328	RedOx-enzymes	211	AA1_1 Laccase 2 (poxa3)	628	460								0	0								S (0.998/ 20)	57725
PoPC9FM116143	RedOx-enzymes	211	AA1_1 Laccase 2 (poxa3)	628	460			322	543				0.8	0.56			0.22	0.41				S (0.998/ 20)	57780
PoPC15FM1089723	RedOx-enzymes	212	AA1_1 Laccase 10 (poxC)	1352									0									S (1.000/ 24)	57046
PoPC9FM81117	RedOx-enzymes	212	AA1_1 Laccase 10 (poxC)	1352			759						0.49			0.22						S (1.000/ 24)	57016
PoPC15FM1048750	RedOx-enzymes	213	AA3_2 Put. aryl-alcohol oxidase			115									0							S (0.999/ 22)	62544
PoPC9FM59433	RedOx-enzymes	213	AA3_2 Put. Aryl-alcohol oxidase			115			296						0.14			0.24				S (1.000/ 22)	65052
PoPC15FM1098737	RedOx-enzymes	214	AA3_2 Put. aryl-alcohol oxidase (FAD-dependent, GMC-oxidoreductase)									152									0.14	S (1.000/ 21)	63535
PoPC15FM1038306	RedOx-enzymes	215	AA5_1 Put. copper radical oxidase, fragment			347									0							Q (0.013/ 25)	59023
PoPC9FM94009	RedOx-enzymes	215	AA5_1 Put. copper radical oxidase			395									0.48							Q (0.008/ 25)	58252
PoPC9FM107973	RedOx-enzymes	216	Put. FAD-linked oxidoreductase						137									0				S (1.000/ 20)	54526
PoPC15FM1114567	RedOx-enzymes	217	Soluble quinoprotein glucose/sorbosone dehydrogenase	575	1465	4176					175	874	0.42	0.9	0.69					0.26	0.69	S (1.000/ 17)	48450
PoPC9FM90832	RedOx-enzymes	217	Soluble quinoprotein glucose/sorbosone dehydrogenase	551	1298	4199	1249	5496	13469				0.34	0.6	0.51	0.6	1.27	1.02				S (1.000/ 17)	48343
PoPC15FM1090605	RedOx-enzymes	218	Unknown protein (cupredoxins, put. Redox-proteins)	155						996	914	714	0									S (1.000/ 17)	20596
PoPC9FM79407	RedOx-enzymes	218	Unknown protein (cupredoxins, put. Redox-proteins)	155									0.31									S (1.000/ 17)	20495
PoPC15FM1031770	RedOx-enzymes	219	Unknown protein (2 cupredoxin domains)							285	499	381										S (1.000/ 18)	39152
PoPC9FM43770	RedOx-enzymes	219	Unknown protein (2 cupredoxin domains)				332	303								0.33	0.24					S (1.000/ 18)	39201
PoPC15FM1062660	RedOx-enzymes	220	Unknown proteins (cupredoxins)	559		897				671	621	1143	0.38		0				0.47	0.57		S (0.999/ 20)	44013
PoPC9FM71620	RedOx-enzymes	220	Unknown proteins (cupredoxins)	550		897	356						0		0.67	0.21						S (0.999/ 20)	44017
PoPC15FM1082678	Unknown function	221	Cyanovirin-N-like protein		226									0.59								S (1.000/ 25)	11434
PoPC15FM1113759	Unknown function	222	Hemopexin family-like protein							169												Q (0.000/ 22)	26582
PoPC9FM86351	Unknown function	223	Put. RHS-repeat protein (annotation problem?)			413									0.07							Q (0.000/ 33)	222301
PoPC15FM1097844	Unknown function	224	Ricin B/Lectin like protein, carbohydrate binding	499	1032	2037				1097	699	2744	0	0	0							S (1.000/ 21)	45446
PoPC9FM116255	Unknown function	224	Ricin B/Lectin like protein, carbohydrate binding	499	1032	2037	230		415				0.37	0.75	0.65	0.21		0.28				S (1.000/ 21)	45422
PoPC15FM1095211	Unknown function	225	Thaumatin-like protein	220									0									S (0.999/ 18)	27898
PoPC9FM109482	Unknown function	225	Thaumatin-like protein	220									0.65									S (0.999/ 18)	27898
PoPC15FM1100408	Unknown function	226	Unknown protein		318									0								S (1.000/ 19)	24508
PoPC9FM128249	Unknown function	226	Unknown protein		318									0.4								S (1.000/ 19)	24510
PoPC15FM1089609	Unknown function	227	Unknown protein	222	295	161				239	1774	1681	0	0	0							S (0.993/ 25)	14135
PoPC9FM115427	Unknown function	227	Unknown protein	222	295	161			418				1.15	1.15	0.78			1.15				S (0.993/ 25)	14135
PoPC15FM1036945	Unknown function	228	Unknown protein			466					455				0							Q (0.009/ 16)	27501
PoPC9FM86238	Unknown function	228	Unknown protein			466			579						0.3			0.7				S (0.999/ 19)	31644
PoPC15FM1045423	Unknown function	229	Unknown protein (put. Expansin-like, missing SP)		587	415				483	720	329		1.53	2.67					2.05	1.53	Q (0.000/ 15)	14525

proteinaccession	ShortName	Allelic pair	ProteinName	dkN001			mkPC9			mkPC15			dkN001			mkPC9			mkPC15			SignalP	prot_mass
				W	WG	G	W	WG	G	W	WG	G	W	WG	G	W	WG	G	W	WG	G		
PoPC9FM87454	Unknown function	229	Unknown protein (put. Expansin-like)		587	415			421					0	0			0				S (1.000/ 21)	16108
PoPC15FM41292	Unknown function	230	Unknown protein (put. glycosyl hydrolase)		306					197	214			0.27					0.18	0.27		S (0.999/ 18)	34714
PoPC9FM86577	Unknown function	230	Unknown protein (put. glycosyl hydrolase)		321									0.38								S (0.999/ 18)	34698
PoPC15FM1089134	Unknown function	231	Unknown protein (put. GPI-anchored)			762				129	531	770			0							S (1.000/ 19)	19893
PoPC9FM69993	Unknown function	231	Unknown protein (put. GPI-anchored)			762	233		302						0.74	0.74		0.74				S (1.000/ 19)	19877
PoPC15FM1066640	Unknown function	232	Unknown protein (put. GPI-anchored)		398					574	409			0.38					0.54	0.54		S (0.999/ 19)	25734
PoPC9FM72871	Unknown function	232	Unknown protein (put. GPI-anchored)		372			403						0			0.24					S (0.999/ 19)	25737
PoPC15FM1089988	Unknown function	233	Unknown protein (putative carbohydrate binding protein)	474	441	975				1682	2628	1460	0	0	0							S (1.000/ 25)	40839
PoPC9FM117301	Unknown function	233	Unknown protein (putative carbohydrate binding protein)	474	441	975	590		1216				0.41	0.41	0.41	0.41		0.62				S (1.000/ 25)	40811
PoPC15FM1067734	Unknown function	234	Unknown protein (putative carbohydrate binding protein)		259	394					819	1218		0.24	0.33					0.43	0.33	S (1.000/ 24)	39138
PoPC9FM116525	Unknown function	234	Unknown protein (putative carbohydrate binding protein)		259	394	521		648					0	0	0		0				S (1.000/ 24)	39196
PoPC15FM1114460	Unknown function	235	Unknown protein (TcsB_toxin/Rhs repeats)	339		1604				548	505	762	0.25		0.54				0.23	0.23	0.32	Q (0.000/ 23)	218708
PoPC9FM110973	Unknown function	235	Unknown protein (TcsB_toxin/Rhs repeats)	338		1516	204						0		0.52	0						Q (0.000/ 23)	212663
PoPC15FM170656	Unknown function	236	Unknown protein (TcsB_toxin/Rhs repeats)			263									0							Q (0.000/ 33)	221309
PoPC9FM96527	Unknown function	236	Unknown protein (TcsB_toxin/Rhs repeats)	257		301							0.13		0.05							Q (0.000/ 33)	219436
PoPC15FM1072880	Unknown function	237	Unknown protein	143							609		0									S (0.999/ 22)	26176
PoPC9FM74882	Unknown function	237	Unknown protein	143									0.38									S (0.996/ 22)	26125
PoPC15FM1087565	Unknown function	238	Unknown protein	671						585	409		0							3.19		Q (0.000/ 24)	11131
PoPC9FM117865	Unknown function	238	Unknown protein	671			743	570					2.58			3.62	3.62					Q (0.000/ 24)	10296
PoPC15FM3756	Unknown function	239	Unknown protein		768					289	522			0								Q (0.000/ 20)	25923
PoPC9FM20650	Unknown function	239	Unknown protein		768				479					0.38				0.24				Q (0.000/ 20)	25923
PoPC9FM65712	Unknown function	240	Unknown protein						782									1.1				S (0.999/ 23)	18448
PoPC9FM64954	Unknown function	241	Unknown protein				422		383							0.27		0.4				S (0.945/ 26)	59732
PoPC15FM1048324	Unknown function	242	Unknown protein									602									0.57	Q (0.003/ 39)	37446
PoPC9FM87860	Unknown function	242	Unknown protein						299									0.22				Q (0.030/ 16)	42705
PoPC15FM1112828	Unknown function	243	Unknown protein									264										S (1.000/ 24)	27085
PoPC9FM115392	Unknown function	243	Unknown protein						166									0.36				S (1.000/ 20)	27102
PoPC15FM1034270	Unknown function	244	Unknown protein							125	186	304										Q (0.000/ 0)	18291
PoPC9FM67491	Unknown function	244	Unknown protein					111									0.35					Q (0.000/ 0)	18291
PoPC9FM128248	Unknown function	245	Unknown protein					881	1206								2.15	1.5				S (0.999/ 19)	24243
PoPC9FM101358	Unknown function	246	Unknown protein (fragment?)				369									0						Q (0.000/ 0)	30594
PoPC9FM51118	Unknown function	247	Unknown protein (possible sequence problem)						1850									0.38				S (0.999/ 26)	105935
PoPC9FM80285	Unknown function	248	Unknown protein (put. hydrophobic surface binding protein)					163	682								0.56	1.81				S (0.999/ 20)	18568
PoPC15FM1111045	Unknown function	249	Unknown protein (put. peptidase)									230									0.26	S (0.999/ 22)	74795
PoPC9FM116865	Unknown function	249	Unknown protein (put. peptidase)						186									0.17				S (0.999/ 22)	73052
PoPC9FM74745	Unknown function	250	Unknown protein, CBM13					463	204								0.88	0.37				S (1.000/ 21)	17346
PoPC15FM1113794	Unknown function	251	Unknown protein	2253	1435					4203	10540	9060	0	0								S (0.999/ 22)	21304
PoPC9FM72289	Unknown function	251	Unknown protein	2253	1435				1022				0.63	0.63				0.63				S (0.999/ 22)	22705
PoPC9FM74226	Unknown function	252	Unknown protein		497	308			370					0.96	0.5			0.31				S (0.999/ 22)	20371
PoPC15FM45648	Unknown function	253	Unknown protein		283						269	216		0						0.28	0.18	S (1.000/ 20)	34198
PoPC9FM37884	Unknown function	253	Unknown protein		283			154						0.21			0.13					S (0.985/ 15)	45298
PoPC15FM1066218	Unknown function	254	Unknown protein (fragment)																0.42			Q (0.017/ 28)	47999
PoPC9FM98428	Unknown function	254	Unknown protein	261						321			0.52									S (1.000/ 24)	54553
PoPC15FM1114553	Unknown function	255	Unknown protein, annotation problem	308									0.12									S (1.000/ 24)	131107
PoPC9FM101357	Unknown function	255	Unknown protein (fragment?)	230			849						0			0						Q (0.018/ 45)	54232
PoPC15FM1033818	Unknown function	256	Unknown protein (fragment?)							548	294											Q (0.000/ 56)	27623
PoPC15FM1065820	Unknown function	257	Unknown protein (put. lectin)							220	1398								1.11	5.5		Q (0.001/ 18)	14458
PoPC15FM1090622	Unknown function	258	Unknown protein								308	469										S (0.999/ 25)	34115
PoPC15FM1072386	Unknown function	259	Unknown protein									325									0.43	S (0.998/ 24)	23261
PoPC15FM1093062	Unknown function	260	Unknown protein									319										S (0.996/ 41)	26359
PoPC15FM172974	Unknown function	261	Unknown protein							637	350								1.67	0.8		Q (0.000/ 0)	28395
PoPC15FM1062047	Unknown function	262	Unknown protein							491												S (1.000/ 21)	38712
PoPC15FM1064711	Unknown function	263	Unknown protein							463												Q (0.000/ 15)	20205
PoPC15FM1090912	Unknown function	264	Unknown protein							180									0.23			S (0.999/ 26)	40935
PoPC15FM1076067	Unknown function	265	Unknown protein									467										Q (0.003/ 19)	44111
PoPC15FM1111409	Unknown function	266	Unknown protein								801	813								0.55	0.34	S (1.000/ 21)	38509
PoPC15FM172626	Unknown function	267	Unknown protein							239	461	295							0.2	0.43	0.2	S (1.000/ 22)	31234
PoPC15FM1066864	Unknown function	268	Unknown protein							199	877	1063										S (0.996/ 21)	34651
PoPC15FM1114137	Unknown function	269	Unknown protein (annotation problem?)								232									0.09		S (0.999/ 23)	68206
PoPC15FM1075358	Unknown function	270	Unknown protein (fragment?)									286									0.43	Q (0.010/ 26)	39062
PoPC15FM1072574	Unknown function	271	Unknown protein (fragment?)							374									1.05			Q (0.000/ 22)	15109
PoPC15FM1062696	Unknown function	272	Unknown protein (put. [illegible]LC-like phosphodiesterase)									832									0.79	S (0.983/ 22)	33752
PoPC15FM1109843	Unknown function	273	Unknown protein (put. chitin binding)							582									0.49			S (1.000/ 20)	20744
PoPC15FM165420	Unknown function	274	Unknown protein (put. hydrophobic surface binding protein)							705	441								3.05	2.52		S (0.997/ 20)	19592
PoPC15FM1094527	Unknown function	275	Unknown protein (put. Involved in cell wall beta-glucan synthesis)							1136		257							0.7			S (0.993/ 27)	20800
PoPC15FM1087559	Unknown function	276	Unknown protein (put. involved in cell wall beta-glucan synthesis)							321												S (0.999/ 18)	21873
PoPC15FM1047638	Unknown function	277	Unknown protein (sequence problem?)									134									0.15	S (0.988/ 24)	62835
PoPC15FM1098210	Unknown function	278	Unknown proteins							296									0.95			S (0.999/ 17)	20640
**278 unique proteins**																							

Supplementary Table 3. Functional classification of proteins identified in the secretomes of dikaryon N001 and monokaryons PC9 and PC15 cultivated in liquid shaken media containing glucose (G), wood (W) or glucose plus wood (WG) as carbon source.

	dkN001			mkPC9			mkPC15		
	**W**	**W+G**	**G**	**W**	**W+G**	**G**	**W**	**W+G**	**G**
Glycoside hydrolases	43	18	11	45	10	10	56	35	31
Unknown function	11	13	11	9	7	19	28	25	24
RedOx-enzymes	11	8	11	10	11	15	9	9	15
Intracellular cont.	7	5	2	4	2	12	19	14	10
Proteases	11	6	8	2	1	11	9	9	14
Esterases	5	2	1	9	4	6	6	6	4
Lyases	8	3	1	8	3	3	8	6	4
Non-Enzyme	3	1	2	0	2	3	8	8	4
Isomerases	2	0	0	1	0	1	2	2	2
Phosphatases	0	1	0	0	0	0	0	0	1
Other hydrolases	1	1	2	0	0	0	2	1	2
Nº of Proteins	**102**	**58**	**49**	**88**	**40**	**80**	**147**	**115**	**111**
